# Mesoporous Poly(melamine-*co*-formaldehyde) Particles for Efficient and Selective Phosphate and Sulfate Removal

**DOI:** 10.3390/molecules26216615

**Published:** 2021-10-31

**Authors:** Konstantin B. L. Borchert, Christine Steinbach, Berthold Reis, Niklas Gerlach, Philipp Zimmermann, Simona Schwarz, Dana Schwarz

**Affiliations:** Leibniz-Institut für Polymerforschung Dresden e.V., Hohe Str. 6, 01069 Dresden, Germany; borchert@ipfdd.de (K.B.L.B.); steinbach@ipfdd.de (C.S.); reis@ipfdd.de (B.R.); gerlach@ipfdd.de (N.G.); zimmermann-philipp@ipfdd.de (P.Z.); simsch@ipfdd.de (S.S.)

**Keywords:** porous resin particles, sorption, oxyanion removal, melamine–formaldehyde resin, hard templating, silica, water treatment, selectivity

## Abstract

Due to the existence-threatening risk to aquatic life and entire ecosystems, the removal of oxyanions such as sulfate and phosphate from anthropogenic wastewaters, such as municipal effluents and acid mine drainage, is inevitable. Furthermore, phosphorus is an indispensable resource for worldwide plant fertilization, which cannot be replaced by any other substance. This raises phosphate to one of the most important mineral resources worldwide. Thus, efficient recovery of phosphate is essential for ecosystems and the economy. To face the harsh acidic conditions, such as for acid mine drainage, an adsorber material with a high chemical resistivity is beneficial. Poly(melamine-*co*-formaldehyde) (PMF) sustains these conditions whilst its very high amount of nitrogen functionalities (up to 53.7 wt.%) act as efficient adsorption sides. To increase adsorption capacities, PMF was synthesized in the form of mesoporous particles using a hard-templating approach yielding specific surface areas up to 409 m^2^/g. Different amounts of silica nanospheres were utilized as template and evaluated for the adsorption of sulfate and phosphate ions. The adsorption isotherms were validated by the Langmuir model. Due to their properties, the PMF particles possessed outperforming maximum adsorption capacities of 341 and 251 mg/g for phosphate and sulfate, respectively. Furthermore, selective adsorption of sulfate from mixed solutions of phosphate and sulfate was found for silica/PMF hybrid particles.

## 1. Introduction

Oxyanions such as sulfate and phosphate are one of the most frequently occurring pollutants in natural water bodies. High levels of sulfate in water cause corrosion of steel and cement in urban sewers, pipes and bridges [1,2]. Sulfate is toxic for various aquatic lifeforms [3,4] and can be microbiologically converted to hydrogen sulfide, leading to additional contamination [5,6]. Sulfate pollution is naturally caused by weathering of minerals or volcanic activity, but the anthropogenic share of pollution by, e.g., mining drainage, coal combustion, sewage infiltration and synthetic detergents increases with progressing industrialization [7,8,9,10]. Phosphate has a drastic effect on the environment, as it represents the growth-limiting factor for most water bodies and is therefore decisive for harmful algal blooms and eutrophication [11,12,13,14,15,16]. This leads, e.g., to oxygen deficiency and fouling mud formation, threatening many aquatic lifeforms [17]. Anthropogenic phosphate pollution is mainly caused by fertilization and soil erosion connected to farming activities as well as wastewater discharge [11,16,18].

Furthermore, phosphorus from rock resources is finite while its demand is increasing rapidly [19] with the growing world population. Therefore, more attention needs to be paid to the recovery of phosphorous from various sources [20,21,22,23,24,25,26] such as sewage sludge [27,28,29], metalworking slag [30,31,32,33] or municipal waste [34]. One of the most promising solutions is the adsorption from aqueous media such as effluents from leaching processes or from mine drainage [21,22]. However, the low pH values of these solutions, which lead to a corrosive environment, are suggested unsuitable for different types of adsorber materials such as, e.g., various unmodified metal oxides [35,36,37] or biobased adsorbents as unmodified chitosan [38]. For example, different iron oxide and hydroxide species were investigated for the adsorption of phosphate, mainly from solutions with pH ≥ 2 [39,40,41] or even buffered to pH = 7 [42]. When pH is lowered further below a pH of 2.0, the adsorbent is dissolving. Non-modified chitosan as another example is known to be soluble in highly acidic media, making it unsuitable for the adsorbent in acid wastewaters. Poly(melamine-*co*-formaldehyde) (PMF) is a chemically extremely resistant polymer resin with a high amount of nitrogen functionalities, which makes the material a suitable adsorber at all pH values. Thus, in the last decade various synthetic concepts were developed to obtain a highly nanoporous material with a moderate monodisperse particle size. One of the approaches comprises heating of the reaction solution in DMSO in closed vessels up to 180 °C yielding in DMSO decomposition and sulfur residuals in the polymer [43,44,45]. Another concept to obtain spherical nanoporous PMF particles involves emulsion polymerization in Span^®^80 and n-dodecane [46]. The efficient adsorption of heavy metal ions such as Pb^2+^, methylene blue and sulfate on PMF has already been reported [45,46,47,48,49]. To the best of our knowledge, the adsorption properties of PMF toward sulfate and phosphate have not been investigated, with the exception of our author team [47]. The adsorption capacities from phosphate and sulfate were not yet specified by application of isotherm models. Furthermore, recycling of lithium-based batteries often only aims for the recovery of Li and other metals, while phosphate from, e.g., lithium iron phosphate or LiPF_6_ batteries is of lesser interest. In the recycling processes, hydrometallurgical processes are often applied. This includes oxidation with, e.g., H_2_SO_4_ and H_2_O_2_ or Na_2_S_2_O_8_ and acid leaching of the components, or extraction with phosphoric acid [50,51,52], which leads to highly acidic and phosphorous rich waste streams often also containing high levels of sulfate. Here, a feasible P-recovery procedure could be a selective separation achieved by an adsorption process.

Here, we present a simple and purely water-based green concept for the synthesis of mesoporous PMF particles, using silica nanoparticles (SiO_2_ NPs) as a hard template. We investigated the impact of the SiO_2_ NPs (66 to 89 wt.%) as a hard template for monodisperse mesopores and on the particle formation acting as Pickering emulsion. Hence, we characterized the particles with both scanning and transmission electron microscopy, laser diffractometry and nitrogen sorption to determine the particle size distribution and pore size distribution (PSD), respectively. These results were used to gain insights into the pore and particle formation mechanism. Further, the chemical structure was investigated using ATR-FTIR spectroscopy and elemental analysis to examine the differences in chemical composition. Finally, the characterized particles were used in adsorption experiments with sulfuric and phosphoric acid. The obtained isotherms were validated by Langmuir and Dubinin–Radushkevich isotherm models. Furthermore, the PMF particles were investigated for selective sulfate and phosphate adsorption.

## 2. Results and Discussion

A hard templating approach with monodisperse SiO_2_ NPs (12 nm in diameter) was applied for the synthesis of mesoporous PMF particles. To investigate the influence of the SiO_2_ NPs on the particle and pore formation, the amount of template (66 to 89 wt.%) for the PMF particle synthesis was varied. Hence, the labeling of the samples includes a suffix referring to the theoretical initial silica content in wt.% in the hybrid material. The prefix H- corresponds to the silica hybrid particles without removal of the template and the prefix P- corresponds to the pure PMF particles after removal of the template.

### 2.1. Synthesis and Characterization of the PMF Particles

The chemical structure of the PMF particles was analyzed by ATR-FTIR, EA and TGA. ATR-FTIR measurements featured a broad band between 3280 cm^−1^ and 3500 cm^−1^ originating from various valence modes of secondary amines (Figures S1 and S2). The more discrete band at 2956 cm^−1^ was attributed to methylene stretching. Together with the bands at 1487 and 1350 cm^−1^ assigned to CH_2_ bending, these bands confirmed the formation of methylene bridges. The rather prominent band at 1550 cm^−1^ was attributable to both NH bending and C=N valence modes. The less prominent band at around 1000 cm^−1^ originated from the C–O–C ether bridges. Further, the band at 812 cm^−1^ was assigned to the bending of the triazine ring. Since this mode should be constant throughout all samples, it was used for normalization. Every sample spectrum exhibited these characteristic bands matching the literature [53,54,55] and confirming the formation of the melamine resin matrix in all cases. 

**Figure 1:**
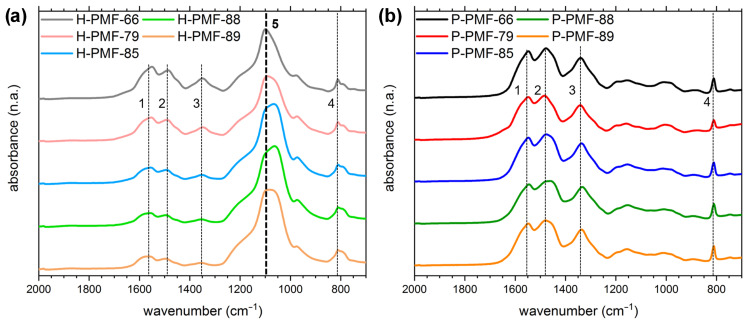
ATR-FTIR spectra of (**a**) H-PMF and (**b**) P-PMF samples. All spectra were normalized to the bending of the triazine ring at 812 cm^−1^ for comparability. The modes marked with a dashed line are 1 NH bending and ν C=N; 2 + 3 CH_2_ bending; 4 triazine bending; **5** ν Si–O.

Additionally, all H-PMF spectra showed a prominent band at approximately 1100 cm^−1^, which was attributed to the Si–O stretching mode (Figure 1, Si–O mode indicated through bold line). This verified the efficient integration of the silica template into the resin. Further, the absence of this band in the P-PMF spectra proved the thorough removal of the template (compare Figure 1a).

**Figure 2:**
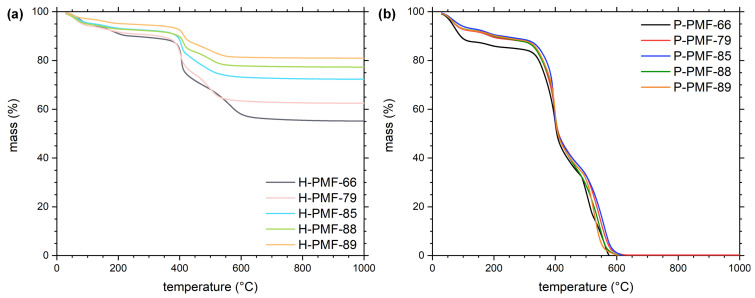
Thermogravimetric analysis from (**a**) the H-PMF samples and (**b**) the P-PMF samples with PMF-66 shown in black, PMF-79 in red, PMF-85 in blue, PMF-88 in green and PMF-89 in orange.

To investigate the successful templating of the PMF particles and the subsequent removal of the silica template in detail, thermogravimetric analysis was conducted. The different ratios of added template to the PMF synthesis can be seen in Figure 2a. At 1000 °C, the hybrid PMF samples exhibited residual masses of 55, 62, 72, 77 and 81 wt.% for H-PMF-66, H-PMF-79, H-PMF-85, H-PMF-88 and H-PMF-89, respectively. As expected, the residual mass increased with the increasing amount of silica added the synthesis. In Figure 2b, no residual mass is presented at 1000 °C, showing the absence of silica. Thus, these results are in agreement with the ATR-FTIR spectra.

The PMF samples exhibited the typical decomposition steps, which are also described in the literature [56,57,58]. Although temperature ranges vary between different publications, the main mass losses are agreed on. First, mainly physical dehydration up to 150 °C can be observed. Second, from 100 to 180 °C, elimination of formaldehyde from free methylolamine groups as well as the condensation of methylolamine groups with each other or amine groups is possible, whereby formaldehyde and water are released. Around 180 to 350 °C, chemical degradation of ether bridges occurred, releasing formaldehyde as a curing process. Starting around 350 °C, methylene bridges degraded and from 390 °C degradation of the triazine ring occurred [56,57,58].

From the TGA results, it can be observed that among all P-PMF samples a very similar decomposition can be found, both in the temperatures of the decomposition steps and also in the mass loss of each step. This indicates a similar chemical structure of the P-PMF samples.

During the polycondensation reaction between melamine and formaldehyde, different reaction steps occur. The resulting PMF resin can feature different amounts of methylene and ether bridges between the triazine units. For a statistical evaluation of the bridging of the PMF resin, the molar ratio of C to N atoms can be discussed (see Table 1). For all PMF samples, the H-PMF materials exhibited higher relative C/N ratios in comparison to the P-PMF samples, since the oxalic acid or oxalate can interact with the silica surface and is therefore still present in the hybrid samples and removed with the template in the P-PMF. Alternatively, the methylol amine functionalities could participate in the resin/silica interaction. These groups will not be converted into ether or methylene bridges and are subsequently hydrolyzed in the washing steps by 1M NaOH solution as methylol amines are prone to hydrolysis in strong basic or acidic media [59,60]. Therefore, it can be concluded that in the prepared hybrid materials, either oxalic acid species or methylol amine groups participate in the PMF/silica interaction, which are then removed during the NaOH washing procedure. Furthermore, it can be seen that all P-PMF samples comprised a very similar C/N ratio.

**Figure 3:**
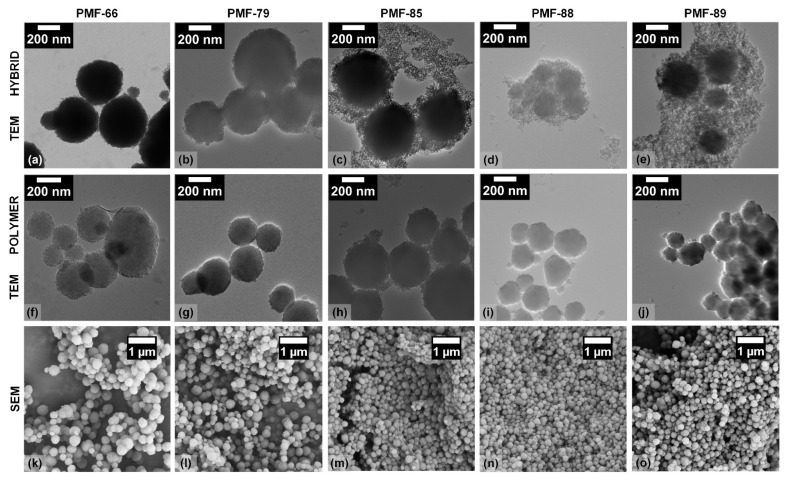
(**a**) TEM image of H-PMF-66, (**f**) TEM image of P-PMF-66 and (**k**) SEM image of P-PMF-66; (**b**) TEM image of H-PMF-79; (**g**) TEM image of P-PMF-79 and (**l**) SEM image of P-PMF-79; (**c**) TEM image of H-PMF-85; (**h**) TEM image of P-PMF-85 and (**m**) SEM image of P-PMF-85; (**d**) TEM image of H-PMF-88; (**i**) TEM image of P-PMF-88 and (**n**) SEM image of P-PMF-88; (**e**) TEM image of H-PMF-89; (**j**) TEM image of P-PMF-89 and (**o**) SEM image of P-PMF-89.

**Figure 4:**
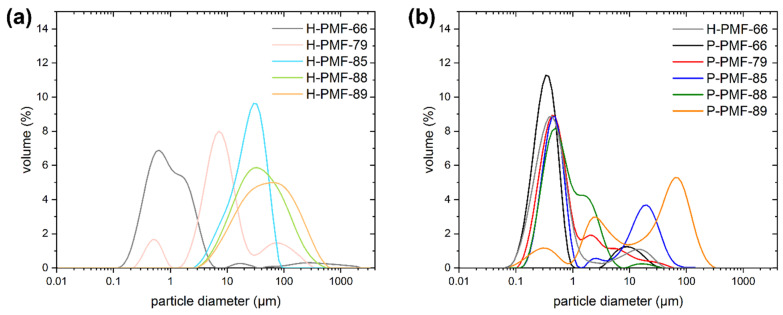
(**a**) Particle size distributions of the unpurified reaction mixtures of H-PMF samples; (**b**) particle size distribution of the P-PMF samples and H-PMF-66 after purification. H-PMF-66 is shown in gray, H-PMF-79 in pink, H-PMF-85 in light blue, H-PMF-88 in light green, H-PMF-89 in light orange. P-PMF-66 is shown in black, P-PMF-79 in red, P-PMF-85 in blue, P-PMF-88 in dark green and P-PMF-89 in orange.

To evaluate the morphology and template inclusion of the H- and P-PMF samples, SEM and TEM investigations were carried out (see Figure 3) as well as particle size measurements (Figure 4). In the last row of Figure 3, the SEM images of the P-PMF samples are presented. For all P-PMF samples, aggregates of spherical primary particles are visible with diameters in the range of hundreds of nanometers. The TEM images of the H-PMF samples display the incorporation of the template (i.e., SiO_2_ NPs) in the PMF resin, which can be seen on the edges of the larger hybrid particles. Additionally, H-PMF-88 and H-PMF-89 shows comparably large amounts of SiO_2_ NPs around the hybrid particles. These silica particles were not noticeably integrated in the PMF resin but instead loose surrounded the H-PMF hybrid particles (especially, e.g., H-PMF-89) due to a poor interaction with PMF resin during hybrid particle synthesis. Two main assumptions can be derived: first, this decreased interaction could be due to the differing pH in the reaction mixtures as presented in Table 1. The increasing amount of alkaline Ludox^®^ HS-40 from PMF-66 to PMF-89 led to an increased pH value of the reaction solution. Thus, the polymerization reaction as well as the interaction between the SiO_2_ NPs and the substrates in the reaction were highly pH sensitive. The isoelectric point (IEP) of the silica NPs was around a pH value of 1.6 [61]. Less surface charge on the SiO_2_ NPs increased aggregation but also the potential to interact with the hydrophobic PMF surface, leading to increased incorporation. Secondly, with the increasing amount of silica, there was potentially not enough PMF in the synthesis to incorporate all SiO_2_ NPs. 

In the TEM images of all P-PMF samples, a complete removal of the silica template can be observed. Furthermore, the remaining pore structure is visible for all samples, leading to a porous particle network and a successful templating approach.

In addition to the electron microscopy images, the particle size distribution curves were determined by dynamic light scattering for the H-PMF reaction mixtures and the purified P-PMF and H-PMF-66 particles (Figure 4). From Figure 4a, it is visible that the reaction mixture of H-PMF-66 and partially H-PMF-79 contained sub-micrometer particles. These can be attributed to smaller silica NP aggregates as well as non-aggregated PMF particles. In contrast, the particle size distribution of H-PMF-85 ranged from 2 to 100 µm and the one of H-PMF-88 and H-PMF-89 only featured a peak between 2 and 600 µm. Therefore, formation of very large aggregates consisting of silica and PMF can be derived, as can also be seen from TEM imaging (Figure 3d,e) and SEM imaging (Figure S3). 

All P-PMF samples possessed bi- or multimodal size distribution with the main peak between 300 and 500 nm except for P-PMF-89 (see Figure 4b). For P-PMF-79 and P-PMF-88 this main peak is relatively broad, featuring a shoulder at larger diameters. P-PMF-89 features three broad peaks at approximately 500 nm, 1.5 µm and the largest at around 70 µm. Comparing the purified H-PMF-66 with P-PMF-66, a minor shift in the particle diameter is visible. This is in agreement with the theory that the outer silica layer is removed by etching with NaOH. In addition, aggregates of hybrid particles that are held together by silica particles are separated in the etching process. 

All samples featured at least one minor peak at larger particle sizes in the µm range, which can be explained by aggregates of the sub-particles. This is also the explanation for the particle size distribution of P-PMF-89: the particle sizes of the spherical sub-particles displayed in Figure 3 are in the range of hundreds of nanometers but large aggregates of them can be seen. The main particle sizes of the other P-PMF particles measured with DLS are in very good agreement with the SEM and TEM images.

**Figure 5:**
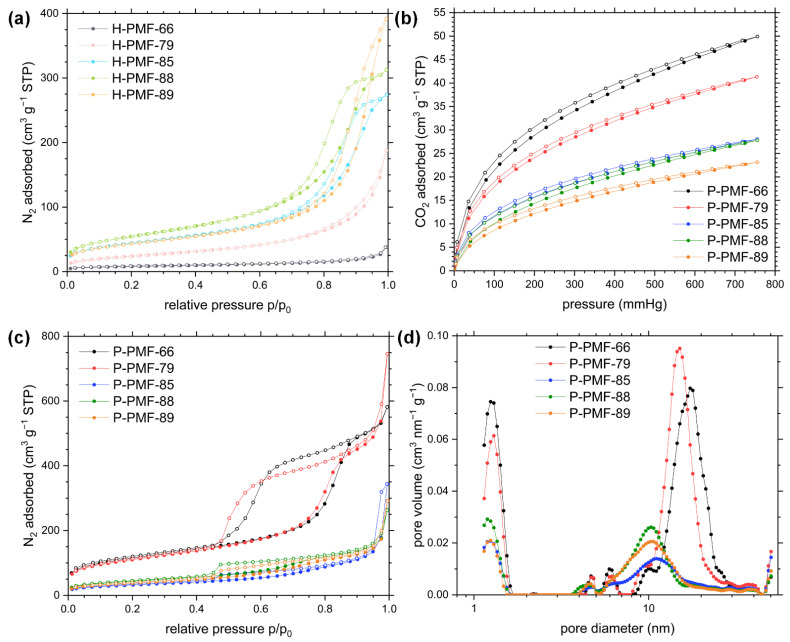
(**a**) Nitrogen (N_2_) de-/adsorption isotherms measured at 77 K for H-PMF samples; (**b**) carbon dioxide (CO_2_) de-/adsorption isotherms measured at 273 K for P-PMF samples; (**c**) nitrogen (N_2_) de-/adsorption isotherms measured at 77 K for P-PMF samples. Datapoints in the adsorption and desorption branch of the isotherms are indicated by filled and empty symbols, respectively. (**d**) Pore size distribution (PSD) analysis for the adsorption branch was calculated by using QSDFT (quenched solid state density functional theory) model for carbon with slit/cylindrical/sphere pores. H-PMF-66 is shown in gray, H-PMF-79 in pink, H-PMF-85 in light blue, H-PMF-88 in light green, H-PMF-89 in light orange P-PMF-66 is shown in black, P-PMF-79 in red, P-PMF-85 in blue, P-PMF-88 in dark green and P-PMF-89 in orange.

To analyze the impact of the SiO_2_ NPs on the porosity of the PMF particles, both materials (hybrid (H) and polymer (P)) were investigated by N_2_ sorption isotherms at 77 K (see Figure 5). The results for the specific surface area, total and micropore volume and CO_2_ uptake are stated in Table 2. The impact of the amount of silica particles can already be seen from the N_2_ sorption isotherms for the hybrid particles (Figure 5a). H-PMF-66 shows a type II isotherm, which is typical for non-porous materials. The other H-PMF samples with higher silica contents featured an isotherm of type IV (H1) character, indicative of large mesopores [62,63]. Furthermore, with an increasing amount of silica particles, the hysteresis is more pronounced yielding higher specific surface areas. Pure spherical silica particles of Ludox^®^ HS-40, washed with EtOH and subsequently dried, exhibited a specific surface area of 185 m^2^/g (see isotherm and PSD in Figures S4 and S5; S_BET_ in agreement with the literature [64,65]). The observations of the PDSs are well in line with previous results obtained by PMF–silica mixtures [46,47].

The P-PMF samples exhibited type IV isotherms with type H1 hysteresis loops starting at p/p_0_~0.42. For type H1 isotherms, adsorption and desorption isotherms are parallel to each other due to an accessible and well-connected pore system. Hence, the P-PMF samples featured ordered mesopores due to the successful implementation of the silica template to the resin. The specific surface area of the P-PMF series had its maximum value at 409 m^2^/g with a silica template content of 66 wt.% (i.e., P-PMF-79, see Table 2, Figure 5c). P-PMF-85 featured the lowest specific surface area with 116 m^2^/g. The PSDs of P-PMF-66 and P-PMF-79 were very similar with pore sizes of around 15 nm in diameter, indicating a well-implemented templating process. P-PMF-85 featured a smaller amount of mesopores yielding in a decrease in surface area. Overall, the peaks of the PSD of P-PMF-85, P-PMF-88 and P-PMF-89 exhibited a generally broader PSD in comparison to P-PMF-79 and P-PMF-66. As already shown in Figure 3, a template amount ≥ 85 wt.% leads to a partial incorporation of SiO_2_ NPs into the polymer network because the ratio of SiO_2_ NPs in comparison to the available amount of PMF is too high. Thus, the broad and smaller PSD of P-PMF-85, P-PMF-88 and P-PMF-89 can be explained by a partial pore collapse in the PMF particles due to the lack of PMF during the synthesis. The network is not as crosslinked and stable as for the samples P-PMF-79 and P-PMF-66. This results in a great variance in pore sizes and a decrease in surface area. 

The CO_2_ sorption experiments and the calculated CO_2_ uptake follows the trend shown by the N_2_ sorption experiments (Table 2, Figure 5d). The relatively high CO_2_ uptake of up to 2.23 mmol/g for P-PMF-66 and the course of the curve indicates a combination of micro- and mesopores in the PMF structure. Furthermore, all samples exhibited hysteresis loops in the adsorption/desorption curve at 273 K.

To further analyze the potential adsorption properties resulting from the chemical structure, streaming potential vs. pH curves were determined for all H- and P-PMF samples, as presented in Figure S6. While the H-PMF samples exhibited an isoelectric point (IEP) between pH 3.7 and 4.8, the IEP of the P-PMF samples featured IEP above pH 6. The positive charge of all PMF samples was due to their abundant amino functionalities, which are partially positively charged in dependence of the pH value. SiO_2_ NPs are negatively charged with an IEP often below a pH of 3.0 [65,66]. Thus, this led to a significant decrease in the IEP values of the H-PMF particles in comparison to the P-PMF particles. 

### 2.2. Sorption Experiments

**Figure 6:**
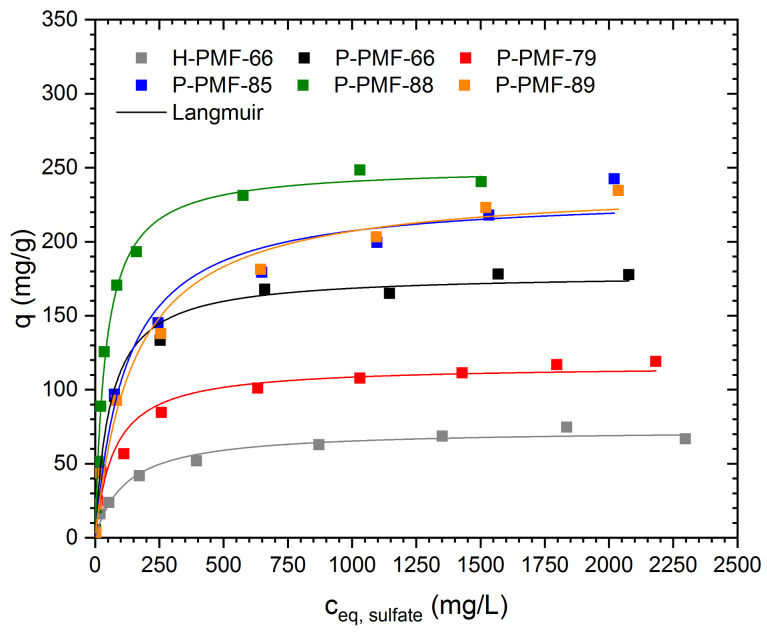
Sorption isotherms for sulfate ions onto H-PMF-66 (gray), P-PMF-66 (black), P-PMF-79 (red), P-PMF-85 (blue), P-PMF-88 (green) and P-PMF-89 (orange) with the corresponding Langmuir fits (solid lines). The corresponding pH values are displayed in Figures S7–S12. The fitting parameters are displayed in Table 3. The respective fitting comparison can be seen in Figure S13.

To investigate the adsorption of oxyanions onto the PMF resins in dependency of the silica content in the materials synthesis, adsorption isotherms of all P-PMF samples with sulfuric acid and phosphoric acid were determined. For comparison, the adsorption isotherm of the hybrid material H-PMF-66 was investigated as well. Furthermore, the isotherms were validated with the Langmuir and Dubinin–Radushkevich model (Figure 6). A fitting comparison is shown in Figure S13.

The adsorption capacity of sulfate ions was determined by increasing the amount of sulfuric acid in solution (i.e., c_0_ increases). Thus, since the pH was not adjusted, the pH decreased with the increasing concentration of sulfate ions. Among all P-PMF samples, P-PMF-88 exhibited the largest experimental adsorption capacity for sulfate with 250 mg/g. The other P-PMF samples showed respective adsorption capacities around 230 mg/g (P-PMF-85 and P-PMF-89), 150 mg/g (P-PMF-66) and 100 mg/g (P-PMF-79). Although P-PMF-66 and P-PMF-79 featured the highest surface areas, both samples possessed the lowest adsorption capacities. P-PMF-88, P-PMF-89 and P-PMF-85 shared relatively similar PSDs with a broad peak between approximately 6 and 14 nm in diameter (Figure 5d), which is beneficial for the adsorption of sulfate ions. In contrast, P-PMF-66 and P-PMF-79 exhibited a monodisperse pore size with diameters around 15 and 17 nm, respectively. The maximum experimental adsorption capacity of the hybrid H-PMF-66 sample was 75 mg/g, which can be explained by the absence of pores and therefore the significantly smaller surface area of the sample. Furthermore, the adsorption capacity for H-PMF-66 was calculated to the total mass of the particles including the 55 wt.% of silica. The Langmuir fits describe the corresponding adsorption isotherms successfully (R^2^ values ranging from 0.979 to 0.995) (see Table 3). Thus, the removal of sulfate ions occurs as monolayer adsorption process on an energetically homogeneous surface. From these results, two main points can be deduced for the adsorption of sulfate: first, the calculated values for the specific surface area by BET from N_2_ sorption measurements do not correlate with the sulfate adsorption capacities for P-PMF. Second, the broad pore size distributions featuring in total smaller pore sizes have a positive effect on the adsorption performance for P-PMF-88, P-PMF-85 and P-PMF-89 (Figure 5d). P-PMF-66 and especially P-PMF-79 featured a very homogenous pore size of approximately 15 nm and significantly lower adsorption capacities. The fitting with Dubinin–Radushkevich showed high R^2^ values, which were below the ones from Langmuir fitting in all cases. Nonetheless, the calculated energy of adsorption values E_Ads_ ranging from 2.99 to 1.38 conclude a physisorption process. Additionally, a strong interaction toward sulfate ions can be deduced from the steep slope of the isotherms at low c_eq_ values and E_Ads_, especially for P-PMF-88, P-PMF-79 and P-PMF-66.

The pH_0_ and pH_eq_ values of the adsorption experiments are shown in Figures S7–S12. For the adsorption mechanism, the pH of the solution is an important parameter. The pH increases through the adsorption process, which can be explained by the protonation of different nitrogen functionalities within the PMF particles enabling ionic interactions to the sulfate ions. Above a pH of 2.0, sulfate is almost to 100% present in its divalent SO_4_^2−^ form. At lower pH values, it shifts to the protonated HSO_4_^−^ form, which is in equimolar share at approximately pH = 1.75 [67]. 

**Figure 7:**
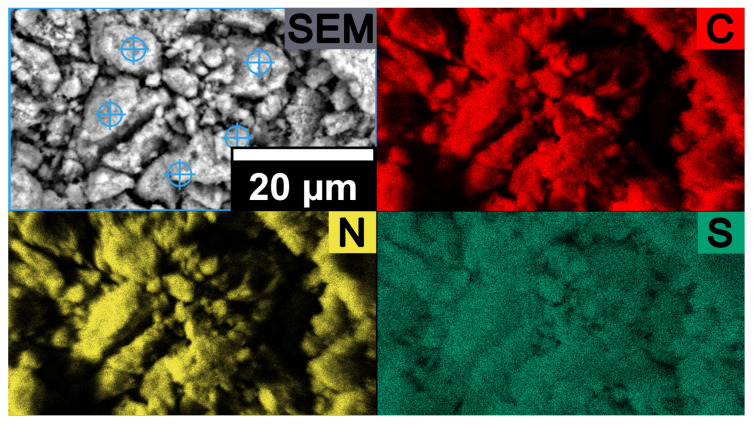
SEM image and SEM-EDX elemental mapping of P-PMF-88 after the adsorption of sulfate with c_eq_ = 1502 mg/g SO_4_^2-^ with the elements C shown in red, N in yellow and S in dark green.

P-PMF-88 with c_eq_ = 1502 mg/L SO_4_^2−^ was further investigated after the adsorption process by SEM-EDX. Figure 7 features a homogeneous distribution of sulfur (i.e., sulfate) on the sample. Thus, the adsorption process occurred on a homogenous surface.

**Figure 8:**
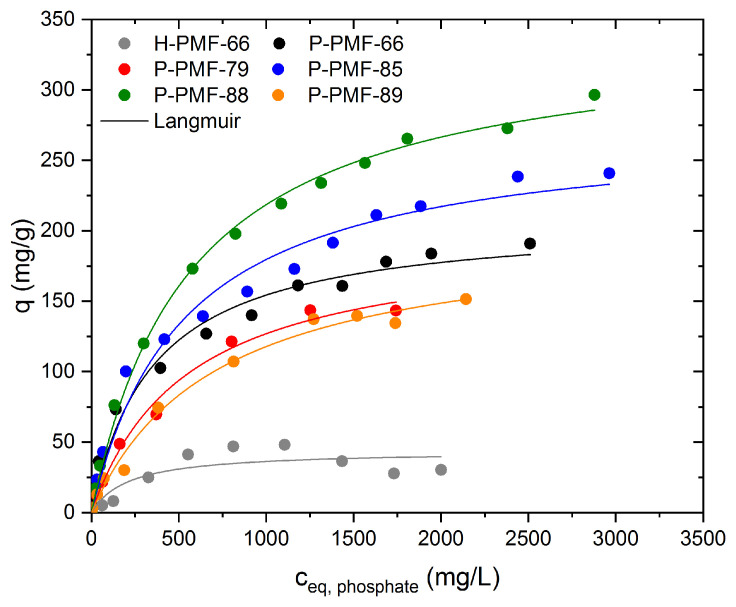
Sorption isotherms for phosphate ions onto H-PMF-66 (gray), P-PMF-66 (black), P-PMF-79 (red), P-PMF-85 (blue), P-PMF-88 (green) and P-PMF-89 (orange) with the corresponding Langmuir fits (solid lines). The corresponding pH values are displayed in Figures S7–S12. The fitting parameters are displayed in Table 4. The respective fitting comparison can be seen in Figure S14.

Additionally, the adsorption of phosphate ions was investigated in the same concentration range as for sulfate ions (Figure 8 and Table 4). Similar to the experiments with sulfate, P-PMF-88 showed the highest adsorption capacity with 297 mg phosphate/g. The second-highest adsorption capacity was achieved for P-PMF-85 (241 mg/g), followed by P-PMF-66 (191 mg/g), P-PMF-89 (151 mg/g) and P-PMF-79 (144 mg/g). Comparing the H-PMF-66 hybrid sample with the corresponding P-PMF-66, the drastic decrease in the adsorption capacity to approximately 45 mg/g is obvious. This effect can be explained by the significantly lower surface area. The trend for the adsorption capacities for phosphate is relatively similar to sulfate except for P-PMF-89.

Comparing the adsorption of sulfate and phosphate, phosphate possessed a significantly higher adsorption capacity despite the higher valency of the phosphate species at low pH values [67,68,69]. In the adsorption experiments, pH_eq_ values between 2.0 and 3.0 were measured for higher phosphate concentrations (see Figures S7–S12). In this pH range, phosphate is present either as monovalent H_2_PO_4_^−^ or uncharged H_3_PO_4_, whereas the concentration of HPO_4_^2−^ or PO_4_^3−^ is negligible. In general, phosphoric acids are less acidic than sulfuric acid, which can alter the adsorption behavior of the samples in terms of ionic interactions due to protonated nitrogen functionalities. Furthermore, phosphate ions and their protonated species are more likely to form hydrogen bonds as its P–O or P=O bonds exhibit a greater dipole moment than the S–O or S=O bonds of sulfate, respectively. Thus, the higher adsorption capacity of phosphate can be explained by the formation of hydrogen bonds between, e.g., N–H and the oxygen of phosphate or an unprotonated nitrogen atom with an O–H group of the phosphate species. In addition, the undissociated H_3_PO_4_ molecule can interact by hydrogen bonding with the PMF polymer. Additionally, the monovalent phosphate species can adsorb by ionic interactions with the PMF particles. Thereby, only one amino group would be needed for this binding motive, leading to higher adsorption capacities. Additionally, as seen in literature for ion exchange materials, a possible process is multiple bound molecules of higher charge sharing binding sites with species of lower charge in order to equalize the charge of both molecules [70]. This would lead to an overall higher adsorption, supporting our findings. The Langmuir and Dubinin–Radushkevich fittings are displayed in Figure S14. Except from H-PMF-66, the Langmuir isotherm model comprised a more valid fitting for the given isotherms with regard to R^2^ values (see Table 4). However, the obtained adsorption capacities from Langmuir showed values around the highest experimental values. The fitting parameters support the outstanding adsorption capacity of P-PMF-88 for phosphate with Q_m_ = 341 mg/g. followed by P-PMF-85 with 275 mg/g. 

The E_Ads_ values for the phosphate adsorption from Dubinin–Radushkevich are in the range of 0.89 to 0.50 kJ/mol. When comparing these with the ones obtained in the adsorption experiments with sulfate, a significantly stronger adsorption interaction for sulfate can be seen which can be, e.g., due to the lower valence of phosphate in this pH region. 

**Figure 9:**
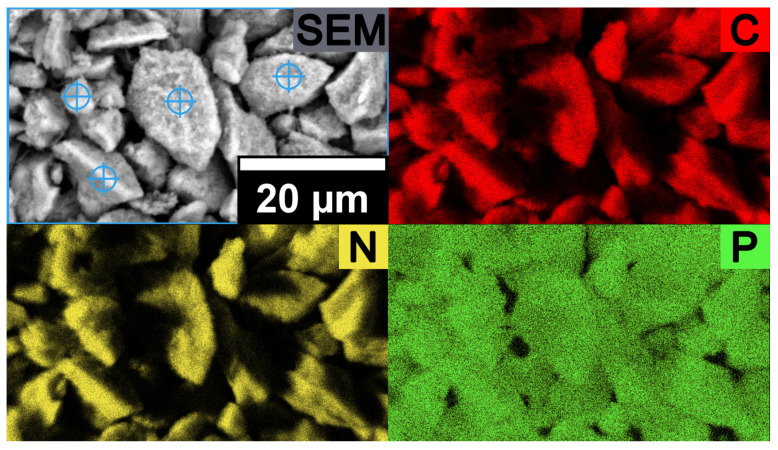
SEM image and SEM-EDX elemental mapping of P-PMF-88 after adsorption of phosphate with c_eq_ = 1502 mg/g PO_4_^3−^ with the elements C shown in red, N in yellow and P in light green.

After the adsorption experiment of the H-PMF-88 sample with phosphate solution (c_eq_ = 2879 mg/L), SEM-EDX analysis for an elemental mapping was carried out (see Figure 9). Similar to sulfate, phosphate is homogeneously distributed over the sample surface which supports the suggestion of a homogenous adsorption process.

The obtained adsorption capacities for phosphate and sulfate outperform different commercially available ion exchange resins which are applied in industrial scale for water treatment applications (see Tables S1 and S2) by far. E.g., Amberlite IRA-900 or Lewatit K6362 as anion exchange resins featured adsorption capacities of 57 and 167 mg sulfate/g, respectively [71,72]. The adsorption capacity of P-PMF was as high as 251 mg/g. For phosphate, adsorption capacities between 24 to 147 mg/g were found for commercially available anion exchange resins [73,74,75]. P-PMF samples here reached outstanding 341 mg/g as adsorption capacities.

**Figure 10:**
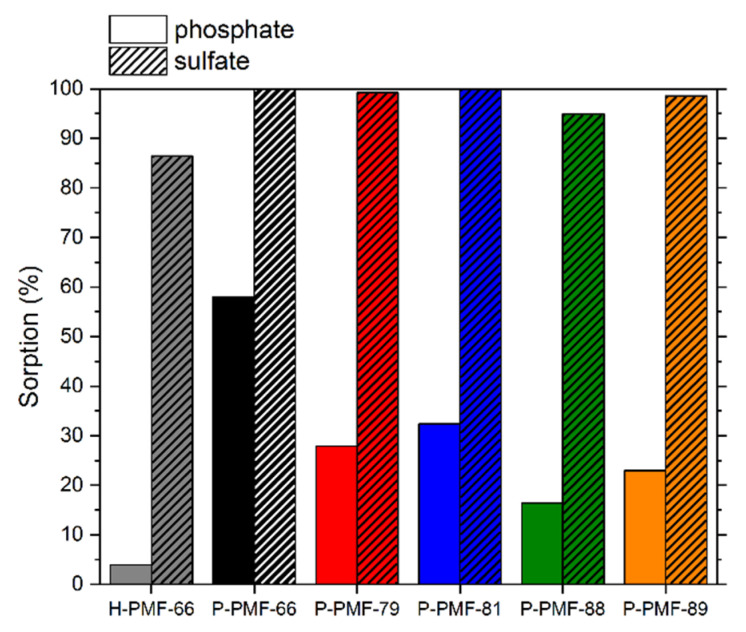
Percentage adsorption of PO_4_^3−^ (solid) and SO_4_^2−^ (striped) for H-PMF-66 and the P-PMF samples from a solution containing both 10 mg/L PO_4_^3−^ and 10 mg/L SO_4_^2−^, whereby H-PMF-66 is shown in gray, PMF-66 in black, P-PMF-79 in red, P-PMF-85 in blue, P-PMF-88 in green and P-PMF-89 in orange. The respective pH_0_ and pH_eq_ values can be seen in Figure S15.

To investigate the adsorption affinity of sulfate in competition with phosphate, adsorption experiments with a solution containing both 10 mg/L sulfate and 10 mg/L phosphate were carried out. The results can be seen in Figure 10 and the corresponding pH values are presented in Figure S15. 

In general, all samples featured a higher adsorption of sulfate than phosphate. The P-PMF samples successfully removed nearly all sulfate ions in the solution (95 to 100%). The simultaneous adsorption of phosphate ions yielded in broad distribution ranging from 16 to 58% of adsorption. Thus, the ratio of adsorbed sulfate per adsorbed phosphate ranged from 1.7 to 5.7 among the P-PMF samples. P-PMF-89 featured the highest adsorption of phosphate with 58%, while at the same time 100% of sulfate was adsorbed as well. This is in agreement with the significantly lower E_Ads_ values for phosphate compared to sulfate, as was shown by the Dubinin–Radushkevich isotherm fitting.

In comparison to the P-PMF samples, H-PMF-66 featured 87% adsorption of sulfate and 4% adsorption for phosphate due to the extremely low surface area. However, the different adsorption process on the surface of the particle led to a ratio of adsorbed sulfate to adsorbed phosphate of approximately 21.6:1, which indicates an exceptional preferable adsorption of sulfate ions. In contrast, the adsorption of phosphate onto the P-PMF samples was significantly higher with a ratio of only 1.7 SO_4_^2−^:PO_4_^3−^. 

**Figure 11:**
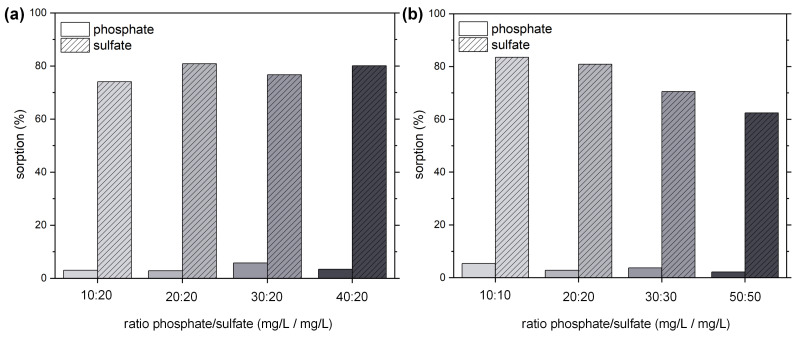
Percentage adsorption of PO_4_^3−^ (solid) and SO_4_^2−^ (striped) for H-PMF-66 from (**a**) solutions containing both phosphate (at different concentrations) and 20 mg/L sulfate; (**b**) solutions of 1:1 ratios of phosphate and sulfate at different concentrations. The respective pH_0_ and pH_eq_ values can be seen in Figures S16 and S17.

As H-PMF-66 showed an outstanding selectivity for sulfate adsorption, we additionally investigated its adsorption behavior for various other phosphate/sulfate ratios and concentrations (Figure 11). The results show a clear selectivity towards the adsorption of sulfate and only minor adsorption of phosphate even in a 40:20 ratio of phosphate to sulfate of the initial solution. Hereby, sulfate ions were adsorbed via ionic interaction on the outer surface of the hybrid particles. Probably, as hydrogen bonds in confined pores could be decisive for phosphate adsorption, the absence of a viable pore structure and the lower strength of hydrogen bonds vs. ionic interactions hindered its adsorption. When increasing the overall concentration while maintaining the 1:1 ratio (Figure 11b), sulfate was still strongly favored by the H-PMF-66 sample, decreasing overall adsorption of both sulfate and phosphate in most cases. As the synthesis of H-PMF-66 is simple, it represents a viable separator for wastewaters contaminated with mixtures of sulfate and phosphate.

## 3. Materials and Methods

### 3.1. Materials

#### 3.1.1. PMF Particles

For the synthesis of the PMF particles, melamine (Sigma-Aldrich, München, Germany, 99%), paraformaldehyde (Sigma-Aldrich, München, Germany, 95%), oxalic acid (Sigma-Aldrich, München, Germany, 99%), NaOH (Honeywell, Offenbach, Germany, ≥98%) and Ludox^®^ HS-40 (Sigma-Aldrich, München, Germany, 40 wt.% in H_2_O) were used as received without further purification. The synthesis was carried out in ultrapure water purified by a Milli-Q Advantage A10^®^ system (Millipore, Darmstadt, Germany) (total organic carbon = 5 ppb, resistivity of 18.2 MΩ∙cm at 25 °C).

#### 3.1.2. Oxyanions

For the adsorption experiments, H_2_SO_4_ (Acros, 96% in H_2_O) and H_3_PO_4_ (Sigma-Aldrich, München, Germany, 85% in H_2_O) were used as received without further purification.

#### 3.1.3. Ultrapure Water

For all experiments, ultrapure water purified by a Milli-Q Advantage A10^®^ system (Millipore, Darmstadt, Germany) (total organic carbon = 5 ppb, resistivity of 18.2 MΩ∙cm) was used.

#### 3.1.4. ICP-OES Standard Solutions

For the ICP-OES measurements 10,000 mg/L P (Bernd Kraft, Duisburg, Germany) and 9998 mg/L S (Sigma-Aldrich, München, Germany) were used as standard solutions.

### 3.2. Methods

Thermogravimetric analysis (TGA) was performed by using the device 1 Star System from Mettler Toledo, Gießen, Germany. The measurements were carried out with approximately 5 to 8 mg of the sample in a platinum crucible. The investigated temperature range was from 25 to 1000 °C with a heating rate of 10 °C/min, under air atmosphere at a flow rate of 40 mL/min.

Scanning electron microscopy (SEM) was carried out using a SEM Ultra Plus from Carl Zeiss Microscopy GmbH, Oberkochen, Germany. For this purpose, the samples were fixed with double-sided adhesive carbon tape on an aluminum pin sample tray and afterwards streamed with N_2_ to obtain only a thin layer of particles. The samples were then sputtered with 3 nm of platinum using a Sputter Coater SCD050 from Leica Microsystems, Wetzlar, Germany before the investigation started. The measurements were carried out with an acceleration voltage of 3 keV at different magnifications.

Transmission electron microscopy (TEM) was carried out using a Libra 120 device from Carl Zeiss Microscopy GmbH, Oberkochen, Germany. The acceleration voltage was 120 keV. The studied particles were dispersed in ultrapure water and dropped onto a carbon-coated Cu mesh.

Particles sizes of both, the purified particles and the unpurified reaction mixtures after synthesis were analyzed using a Zetasizer Nano ZS (Malvern, Kassel, Germany). Therefore, the reaction mixture was prepared as stated in Section 3.3 for each sample. After the synthesis, the dispersion was stirred for 15 min before measurement. Data were evaluated using Particle RI: 1.5, Abs.: 0.1000 and Dispersant RI: 1.3300.

Nitrogen and CO_2_ sorption measurements were performed using the Autosorb iQ MP from Quantachrome Instruments, Boynton Beach, FL, USA. Samples of 100 mg were activated by degassing in vacuum (5 × 10^−10^ mbar) at 110 °C for 24 h. The nitrogen sorption measurements were performed at 77 K. The surface area was calculated in the relative pressure (*p*/*p_0_*) range from 0.07 to 0.22 by BET method [76]. The pore size distribution (PSD) was determined by a QSDFT (quenched solid density functional theory) model fit of the nitrogen adsorption isotherm, including spherical, cylindrical and slit pores of carbon materials. CO_2_ sorption measurements were performed at 273 K.

Attenuated total reflection infrared spectroscopy (ATR-FTIR) measurements were preformed using a Tensor 27 device equipped with a Platinum ATR module both from Bruker Corporation, Billerica, MA, USA. All samples were measured in dry state with a resolution of 2 cm^−1^ and with 100 scans. The acquired spectra were subjected to atmospheric compensation to remove the rotation bands of water. Further, all spectra were normalized using the triazine ring-bending band at 812 cm^−1^. 

Inductively coupled plasma optical emission spectrometry (ICP-OES) (iCAP 7400 from Thermo Scientific) was used to determine the sulfate and phosphate ion concentrations in simulated water. Thus, four standards were used (Standard 1: S (3000 mg/L), P (3000 mg/L) in 4 wt.% HNO_3_; Standard 2: S (1000 mg/L), P (1000 mg/L) in 4 wt.% HNO_3_; Standard 3: S (500 mg/L), P (500 mg/L) in 4 wt.% HNO_3_; Standard 4: S (100 mg/L), P (100 mg/L) in 4 wt.% HNO_3_). To each sample (8 mL) 2 mL 20% nitric acid was added prior to analysis. Each concentration was determined from threefold measurement. 

Streaming potential vs. pH curves were measured to determine the surface charge of the particles in dependence of the pH value. The particles were characterized by titration to either pH 3 or 9 from the initial pH with the particle charge detector Mütek PCD-04 from the company BTG Instruments GmbH, Wessling, Germany with 0.1 M HCl or 0.1 M NaOH, respectively. For this purpose, 0.16 g particles were added to 100 mL of ultrapure water. The dispersion was homogenized with an ultrasonic bath for 2 h. 

Scanning electron microscope with energy-dispersive X-ray spectroscopy (SEM-EDX): The elemental mapping of the samples after adsorption was carried out using a Phenom XL Workstation from Thermo Scientific (Waltham, MA, USA) with an energy-dispersive X-ray spectroscopy detector (Silicon Drift Detector SDD, thermoelectrically cooled (LN2free), 25 mm^2^ detector active area). The samples were fixed on double-sided adhesive carbon tape on an aluminum pin sample tray. The measurements were carried out in high vacuum mode (*p* = 0.1 Pa) with an acceleration voltage of 10 keV at different magnifications.

pH measurement: The measurement of pH was carried out with the device SevenExcellence from Mettler Toledo (Gießen, Germany) at r.t. 

Centrifugation: The adsorber materials were separated from the supernatant by centrifugation with the device 5804 from Eppendorf (Leipzig, Germany) at r.t. and 10,000 rpm.

Elemental analysis was carried out using a vario MICRO cube from the company Elementar, Langenselbold, Germany.

### 3.3. Synthesis of the PMF Particles

#### 3.3.1. Synthesis of PMF-66

The PMF-66 particles were synthesized as recently published [47] with minor modifications by first dispersing 9.1 g (72.2 mmol) melamine (M) and 12.95 g (431.2 mmol) paraformaldehyde (F) in 175 mL ultrapure water in a 1 L round bottom flask. The dispersion was stirred at 50 °C for 40 min under reflux. A solution of 525 mL ultrapure water with 1.4 g (15.5 mmol) oxalic acid and 42.0 g Ludox^®^ HS-40 was prepared and then added to the reaction mixture. The resulting mixture was stirred for 24 h at 100 °C under reflux.

#### 3.3.2. Synthesis of PMF-79, PMF-85, PMF-88 and PMF-89 Particles

The synthesis of PMF-79, PMF-85, PMF-88 and PMF-89 was modified by increasing the amount of Ludox^®^ HS-40 (see Table 5). PMF particles containing silica particles are called hybrid (H-). After the removal of the silica particles by treatment of the hybrid particles with NaOH, nanoporous PMF-particles (P-) were obtained. 

Purification of H-PMF samples: The sediment was transferred into a 1 L vessel with ultrapure water. The particles were washed in ultrapure water three times. Meanwhile, the vessel with the dispersion was placed on a shaker for 24 h at r.t. Subsequently, the sedimented particles were freeze-dried. As these particles still contained the SiO_2_ NPs, these samples are called hybrid PMF (H-PMF) samples.

Purification of P-PMF samples: To obtain the pure PMF particles, the sediment of the reaction mixture was transferred into a 1 L vessel and filled with 1 M NaOH solution to remove the SiO_2_ NPs. The solution was placed on a shaker for 24 h. The washing procedure with 1 M NaOH was repeated two more times. The particles were then transferred to a Spectra/Por^TM^ 2 dialysis bag (Spectrum Chemical Mfg. Corp., New Brunswick, NJ, USA) and dialyzed with ultrapure water. The resulting particles were freeze-dried.

### 3.4. Water Treatment Experiments

#### 3.4.1. Adsorption Experiments with Sulfuric and Phosphoric Acid 

Next, 50 mg of each adsorber material was placed into a 50 mL centrifuge tube. Subsequently, 30 mL of the adsorptive solution was added to every sample. The pH of the samples was not adjusted. The samples were then magnetically stirred for 24 h at r.t. Afterwards, the samples were centrifuged for 12 min at 10,000 rpm. The supernatant of the samples after the adsorption and the initial concentrations of the adsorptive solutions were analyzed by ICP-OES. The pH of the solutions were determined.

#### 3.4.2. Theoretical Model

To determine the sorption efficiency of the PMF samples, the concentrations of the adsorbed oxyanions in equilibrium were detected by ICP-OES. In Equation (1), these concentrations were used for the calculation of the adsorption in percent. Thereby, c_0_ is the concentration of the respective ion in the initial solution and c_eq_ is the concentration after reaching equilibrium.

(1) $$\mathrm{adsorption}\;=100\%\cdot \frac{c_{0}-c_{\mathrm{eq}}}{c_{0}}$$

The respective sorption capacity q_eq_ in equilibrium was calculated as follows:

(2) $$q_{\mathrm{eq}}=\frac{\left(c_{0}-c_{\mathrm{eq}}\right)\cdot V_{L}}{m_{A}}$$

With V_L_ referring to the given volume of the adsorptive solution and m_A_ to the mass of the sorbent material used in the experiment. 

To model the sorption process, Langmuir (Equation (3)) [77] and Dubinin–Radushkevich [78] (Equations (4)–(7)) isotherm models were chosen.

(3) $$q_{\mathrm{eq}}=\frac{Q_{m}\cdot K_{L}\cdot c_{\mathrm{eq}}}{1+K_{L}\cdot c_{\mathrm{eq}}}$$

K_L_ thereby represents the Langmuir equilibrium constant and Q_m_ the maximum adsorption capacity [79,80].

The non-linear equation for the Dubinin–Radushkevich isotherm model is the following [56,57]:

(4) $$q_{\mathrm{eq}}=Q_{m}\cdot \exp\left(-\beta_{\mathrm{DR}}\cdot \varepsilon^{2}\right)$$

Q_m_ again represents the maximum adsorption capacity, β_DR_ the activity coefficient which is related via Equation (7) to the mean free energy of adsorption E_ads,DR_ and ε corresponds to the Polanyi potential, which can also be expressed by Equation (5).

(5) $$\varepsilon =\mathrm{RT}\cdot \ln\left(\frac{c_{S}}{c_{\mathrm{eq}}}\right)$$

c_S_ hereby represents the solubility of the adsorbate. The term inside the logarithm $\frac{c_{S}}{c_{\mathrm{eq}}}$ can be exchanged for $1+\frac{1}{c_{\mathrm{eq}}}$ according to Zhou, leading to the same numerical solution with two requirements: First, this is only possible for values of c_eq_ << c_S_. Second, it is important to use molar concentrations for the fitting [81]. We have implemented both as suggested. This leads to the following term for the Polanyi potential:

(6) $$\varepsilon =\mathrm{RT}\cdot \ln\left(1+\frac{1}{c_{\mathrm{eq}}}\right)$$

(7) $$E_{\mathrm{ads},\mathrm{DR}}=\frac{1}{\sqrt{2\cdot \beta_{\mathrm{DR}}}}$$

## 4. Conclusions

We successfully prepared highly functional PMF samples for use in oxyanion adsorption from simulated wastewater by a facile hard templating method with SiO_2_ NPs. We thoroughly characterized both the hybrid silica/resin samples and the purified samples after template removal. Here, we showed that the preparation of samples with different pore size distributions and different specific surface areas between 136 and 409 m^2^/g is possible. The pore system exhibited a significant influence on the adsorption behavior, whereas the specific surface area did not affect the adsorption capacity as much as expected. P-PMF represent excellent adsorbers for oxyanions with outstanding adsorption capacities of 341 mg PO_4_^3−^/g and 251 mg SO_4_^2−^/g modeled with Langmuir and Dubinin–Radushkevich isotherm model. These adsorption capacities surpass industrially available ion exchange materials by two to five times in terms of adsorption capacity, while at the same time synthesis is possible with very convenient raw materials. The hybrid silica/resin material presents selective adsorption of sulfate, enabling a simple separation technique for wastewaters containing both sulfate and phosphate ions. E.g., in the battery production, wastewaters could first be treated with hybrid silica/PMF particles to adsorb sulfate selectively, and afterwards, separate phosphate with the porous P-PMF particles for phosphate recovery.

## Figures and Tables

**Table 1:** Results of the elemental analysis (C, H, N) for the different PMF samples. The residue describes the relative mass, which is not attributed to the elements C, H and N. *n*/*n* (C/N) is the molar ratio of C atoms/N atoms calculated from elemental analysis.

Sample	C (wt.%)	H (wt.%)	N (wt.%)	Residue (wt.%)	*n*/*n* (C/N)
H-PMF-66	14.6	2.4	19.5	63.5	0.87
P-PMF-66	33.5	4.9	49.4	12.1	0.79
H-PMF-79	11.9	2.2	16.8	69.1	0.82
P-PMF-79	35.6	4.8	52.4	7.1	0.79
H-PMF-85	7.3	1.6	9.9	81.2	0.85
P-PMF-85	35.6	4.8	53.5	6.0	0.78
H-PMF-88	5.7	1.4	7.7	85.3	0.87
P-PMF-88	35.5	4.8	53.7	6.1	0.77
H-PMF-89	5.4	1.2	7.1	86.3	0.89
P-PMF-89	35.3	4.7	53.7	6.2	0.77

**Table 2:** Surface area (S_BET_), pore volume (PV), micro pore volume (MPV) and CO_2_ uptake of the H- and P-PMF samples and dried Ludox^®^ HS-40.

Sample Code	S_BET_ (m^2^ g^−1^) ^a^	PV (cm^3^ g^−1^) ^b^	MPV (cm^3^ g^−1^) ^c^	CO_2_ Uptake (mmol g^−1^) ^d^
H-PMF-66	28	n.a.	n.a.	n.a.
P-PMF-66	409	0.78	0.15	2.23
H-PMF-79	87	0.19	n.a.	n.a.
P-PMF-79	393	0.76	0.15	1.84
H-PMF-85	159	0.39	0.06	n.a.
P-PMF-85	116	0.23	0.04	1.25
H-PMF-88	198	0.45	0.07	n.a.
P-PMF-88	156	0.24	0.06	1.24
H-PMF-89	155	0.50	0.06	n.a.
P-PMF-89	136	0.23	0.05	1.03
Ludox^®^ HS-40 ^e^	185	0.71	0.07	n.a.

^a^ Surface area calculated from N_2_ adsorption isotherm using BET equation; ^b^ pore volume (PV) calculated from N_2_ uptake at p/p_0_ = 0.95; ^c^ micro pore volume (MPV) calculated from N_2_ uptake at p/p_0_ = 0.10; ^d^ CO_2_ uptake calculated for 273 K and 1 bar. ^e^ For Ludox^®^ HS-40 particles precipitated with ethanol and subsequently dried.

**Table 3:** Fitting parameters for Langmuir and Dubinin–Radushkevich isotherm models for sulfate adsorption onto H-PMF-66 and P-PMF samples. Q_m_ thereby is the maximal sorption capacity, K is the equilibrium constant, β_DR_ represents the activity coefficient related to the energy of adsorption E_Ads_. R^2^ (COD) is the coefficient of determination for the respective fit. For all parameters, the corresponding standard error from the fit is given. The respective fitting comparison can be seen in Figure S13.

Sample	Model	Q_m_ mg/g	K L/mg	$\beta_{DR}$ mol^2^/J^2^	E_ads,DR_ kJ/mol	R^2^ (COD)
H-PMF-66	Langmuir	73.0 ± 2.4	8.46 ± 1.39	--	--	0.987
	Dubinin–Radushkevich	64.1 ± 3.5	--	1.56 × 10^−7^ ± 4.7 × 10^−8^	1.79 ± 0.27	0.934
P-PMF-66	Langmuir	178.4 ± 4.2	17.15 ± 2.47	--	--	0.992
	Dubinin–Radushkevich	162.2 ± 7.3	--	6.12 × 10^−8^ ± 1.56 × 10^−8^	2.86 ± 0.36	0.957
P-PMF-79	Langmuir	116.4 ± 4.8	13.67 ± 3.44	--	--	0.968
	Dubinin–Radushkevich	103.22 ± 5.7	--	5.58 × 10^−8^ ± 1.71 × 10^−8^	2.99 ± 0.45	0.917
P-PMF-85	Langmuir	232.4 ± 10.8	8.3 ± 1.96	--	--	0.979
	Dubinin–Radushkevich	205.0 ± 12.4	--	1.96 × 10^−7^ ± 6.83 × 10^−8^	1.59 ± 0.27	0.934
P-PMF-88	Langmuir	250.7 ± 4.5	24.25 ± 1.84	--	--	0.995
	Dubinin–Radushkevich	230.3 ± 6.1	--	5.59 × 10^−8^ ± 5.28 × 10^−9^	2.99 ± 0.14	0.985
P-PMF-89	Langmuir	238.4 ± 8.8	6.61 ± 1.16	--	--	0.987
	Dubinin–Radushkevich	206.5 ± 12.0	--	2.59 × 10^−7^ ± 8.36 × 10^−8^	1.38 ± 0.22	0.940

**Table 4:** Fitting parameters for Langmuir and Dubinin–Radushkevich isotherm models for phosphate adsorption onto H-PMF-66 and P-PMF samples. Q_m_ thereby is the maximal sorption capacity, K is the equilibrium constant, β_DR_ represents the activity coefficient related to the energy of adsorption E_Ads_. R^2^ (COD) is the coefficient of determination for the respective fit. For all parameters, the corresponding standard error from the fit is given. The respective fitting comparison can be seen in Figure S14.

Sample	Model	Q_m_ mg/g	K L/mg	$\beta_{DR}$ mol^2^/J^2^	E_ads,DR_ kJ/mol	R^2^ (COD)
H-PMF-66	Langmuir	43.9 ± 7.6	4.72 ± 3.51	--	--	0.748
	Dubinin–Radushkevich	39.0 ± 3.8	--	7.13 × 10^−7^ ± 3.58 × 10^−7^	0.83 ± 0.21	0.825
P-PMF-66	Langmuir	208.9 ± 9.5	2.83 ± 0.50	--	--	0.984
	Dubinin–Radushkevich	164.2 ± 8.9	--	6.47 × 10^−7^ ± 2.36 × 10^−7^	0.88 ± 0.16	0.907
P-PMF-79	Langmuir	196.4 ± 11.2	1.83 ± 0.28	--	--	0.993
	Dubinin–Radushkevich	138.0 ± 9.7	--	1.16 × 10^−7^ ± 3.42 × 10^−7^	0.66 ± 0.10	0.942
P-PMF-85	Langmuir	274.6 ± 14. 4	1.89 ± 0.33	--	--	0.978
	Dubinin–Radushkevich	204.5 ± 12.2	--	1.19 × 10^−6^ ± 4.42 × 10^−7^	0.65 ± 0.12	0.867
P-PMF-88	Langmuir	341.4 ± 7.3	1.78 ± 0.12	--	--	0.997
	Dubinin–Radushkevich	256.4 ± 12.4	--	1.60 × 10^−6^ ± 4.64 × 10^−7^	0.56 ± 0.08	0.935
P-PMF-89	Langmuir	201.3 ± 12.4	1.41 ± 0.23	--	--	0.990
	Dubinin–Radushkevich	142.6 ± 6.4	--	1.96 × 10^−6^ ± 4.28 × 10^−7^	0.50 ± 0.05	0.964

**Table 5:** Table of reactants in synthesis (melamine (M), paraformaldehyde (F) and Ludox^®^ HS-40 (HS-40)) for of H-PMF and P-PMF particles with the corresponding sample codes.

Sample	m (M)	m (F)	m (HS-40)	Ratio (M-F-HS-40) ^a^	pH (HS-40) ^b^	pH ^c^	pH ^d^
PMF-66	9.1 g	12.95 g	42 g	66%	9.8	2.14	3.52
PMF-79	9.1 g	12.95 g	84 g	79%	9.8	2.44	3.89
PMF-85	9.1 g	12.95 g	126 g	85%	9.8	3.27	4.32
PMF-88	9.1 g	12.95 g	168 g	88%	9.8	4.07	5.01
PMF-89	9.1 g	12.95 g	182 g	89%	9.8	4.15	5.15

^a^ Mass ratio of Ludox^®^ HS-40/(melamine + paraformaldehyde + Ludox^®^ HS-40) in reaction mixture; ^b^ pH of the pure Ludox^®^ HS-40 dispersion; ^c^ pH of the dispersion containing ultrapure water, oxalic acid and Ludox^®^ HS-40 solution; ^d^ pH of the final reaction mixture after 24 h at 100 °C under reflux.

## Data Availability

Data is contained within the article or Supplementary Material.

## Supplementary Materials

The following are available: Figure S1: ATR-FTIR spectra of P-PMF with the predominant mode assignment and normalized to the triazine bending at 812 cm^−1^ for reasons of comparability; Figure S2: ATR-FTIR spectra of H-PMF with the predominant mode assignment and normalized to the triazine bending at 812 cm^−1^ for comparability; Figure S3: SEM images of (a) H-PMF-66, (b) H-PMF-79, (c) H-PMF-85, (d) H-PMF-88 and H-PMF-89; Figure S4: Nitrogen (N_2_) de-/adsorption isotherms measured at 77 K for Ludox^®^ HS-40. Datapoints in the adsorption and desorption branch of the isotherms are indicated by filled and empty symbols, respectively; Figure S5: Pore size distribution (PSD) of H-PMF samples and Ludox^®^ HS-40 (Quenched solid density functional theory fit; slit/cylindrical/sphere pores; adsorption branch). PMF-66 is shown in black, PMF-79 in red, PMF-81 in blue, PMF-88 in green, PMF-89 in orange and Ludox^®^ HS-40 in violet. Main peaks of Ludox^®^ HS-40 PSD marked with dashed gray line for comparison; Figure S6: Streaming potential vs. pH curves of the P-PMF samples with P-PMF-66 (black), P-PMF-79 (red), P-PMF-85 (blue), P-PMF-88 (green) and P-PMF-89 (orange); Figure S7: pH_0_(c_0_) (filled symbols) and pH_eq_(c_eq_) (crossed symbols) points for the adsorption experiments of H-PMF-66 with a) H_2_SO_4_ and b) H_3_PO_4_.; Figure S8: pH_0_(c_0_) (filled symbols) and pH_eq_(c_eq_) (crossed symbols) points for the adsorption experiments of P-PMF-66 with (a) H_2_SO_4_ and (b) H_3_PO_4_; Figure S9: pH_0_(c_0_) (filled symbols) and pH_eq_(c_eq_) (crossed symbols) points for the adsorption experiments of P-PMF-79 with a) H_2_SO_4_ and b) H_3_PO_4_; Figure S10: pH_0_(c_0_) (filled symbols) and pH_eq_(c_eq_) (crossed symbols) points for the adsorption experiments of P-PMF-85 with (a) H_2_SO_4_ and (b) H_3_PO_4_; Figure S11: pH_0_(c_0_) (filled symbols) and pH_eq_(c_eq_) (crossed symbols) points for the adsorption experiments of P-PMF-88 with a) H_2_SO_4_ and b) H_3_PO_4_; Figure S12: pH_0_(c_0_) (filled symbols) and pH_eq_(c_eq_) (crossed symbols) points for the adsorption experiments of P-PMF-89 with (a) H_2_SO_4_ and (b) H_3_PO_4_; Figure S13: Sorption isotherms for sulfate ions onto H-PMF-66 (gray), P-PMF-66 (black), P-PMF-79 (red), P-PMF-85 (blue), P-PMF-88 (green) and P-PMF-89 (orange) with the corresponding Langmuir fits (solid lines) and Dubinin–Radushkevich fits (dashed line). The corresponding pH values are displayed in Figures S7–S12. The fitting parameters are displayed in Table 3. Figure S14: Sorption isotherms for phosphate ions onto H-PMF-66 (gray), P-PMF-66 (black), P-PMF-79 (red), P-PMF-85 (blue), P-PMF-88 (green) and P-PMF-89 (orange) with the corresponding Langmuir fits (solid lines) and Dubinin-Radushkevich fits (dashed line). The corresponding pH values are displayed in Figures S7–S12. The fitting parameters are displayed in Table 4. Figure S15: pH_0_ (solid) and pH_eq_ (striped) values for the phosphate/sulfate selectivity adsorption experiments with a solution containing both, 10 mg/L PO_4_^3−^ and 10 mg/L SO_4_^2−^, whereby H-PMF-66 is shown in gray, PMF-66 in black, P-PMF-79 in red, P-PMF-85 in blue, P-PMF-88 in green and P-PMF-89 in orange.; Figure S16: pH_0_ (solid) and pH_eq_ (striped) values for the phosphate/sulfate selectivity adsorption experiments with a solution containing different concentrations of phosphate and 20 mg/L sulfate onto H-PMF-66 with pH_0_ shown as solid bar and pH_eq_ shown as striped bar; Figure S17: pH_0_ (solid) and pH_eq_ (striped) values for the phosphate/sulfate selectivity adsorption experiments with a solution containing 1:1 ratios of phosphate and sulfate at different concentrations onto H-PMF-66 with pH_0_ shown as solid bar and pH_eq_ shown as striped bar; Table S1: Sorption capacities for the removal of sulfate compounds from aqueous solutions with different sorbent materials and adsorbent doses (a.d.). The obtained sorption capacities from this work were achieved in the batch adsorption experiments; Table S2: Sorption capacities for removal of phosphate from aqueous solutions with different sorbent materials and adsorbent doses (a.d.). The obtained sorption capacities from this work were achieved in the batch adsorption experiments.

## Sample Availability

Samples of the synthesized compounds and of the adsorbents after adsorption are available from the authors.
