# Supplementary material for: Mesoporous Poly(melamine-co-formaldehyde) Particles for Efficient and Selective Phosphate and Sulfate Removal
Source: Molecules. 2021 Oct 31;26(21):6615. doi: 10.3390/molecules26216615 (PMC8588240; doi:10.3390/molecules26216615)
Supplement: Supplementary file 1 [file molecules-26-06615-s001.zip › molecules-1404317-supplementary.pdf]

ELECTRONIC SUPPLEMENTARY INFORMATION

for the manuscript

Mesoporous Poly(melamine-*co*-formaldehyde) Particles for Sulfate and Phosphate Sorption from Simulated Waters

Konstantin B. L. Borchert[1], Christine Steinbach[1], Berthold Reis[1], Niklas Gerlach[1], Philipp Zimmermann[1], Simona Schwarz[1], Dana Schwarz[1*]

[1] Leibniz-Institut für Polymerforschung Dresden e.V., Hohe Str. 6, 01069 Dresden, Germany; borchert@ipfdd.de (K.B.); steinbach@ipfdd.de (C.S.); reis@ipfdd.de (B.R.); gerlach@ipfdd.de (N.G.); zimmermann-philipp@ipfdd.de (P.Z.); simsch@ipfdd.de (S.S.); schwarz-dana@ipfdd.de (D.S.);

* Correspondence: Dana Schwarz, Tel.: +49 351 46 58 542, email: schwarz-dana@ipfdd.de:

Abstract: Due to the existence-threatening risks to aquatic life and entire ecosystems, the removal of oxyanions like sulfate and phosphate from anthropogenic wastewaters, such as municipal effluents and acid mine drainage, is inevitable. Furthermore, phosphorus is an indispensable resource for the world-wide plant fertilization, which cannot be replaced by any other substance. This raises phosphate to one of the most important mineral resources world-wide. Thus, efficient recovery of phosphate is essential for ecosystems and economy. To face the harsh acidic conditions such as for acid mine drainage, an adsorber material with a high chemical resistivity is beneficial. Poly(melamine-co-formaldehyde) (PMF) sustains these conditions whilst its very high amount of nitrogen functionalities act as efficient adsorption sides. To increase adsorption capacities, PMF was synthesized in form of mesoporous particles by using a hard-templating approach. Different amounts of silica nanospheres were utilized as template and evaluated for the adsorption of sulfate and phosphate ions. The adsorption isotherms were validated by the Langmuir model. Due to their properties, the PMF particles possessed outperforming maximum adsorption capacities of 341 mg/g and 251 mg/g for phosphate and sulfate, respectively. Furthermore, selective adsorption of sulfate from mixed solutions of phosphate and sulfate was found for silica/PMF hybrid particles.

Keywords: porous resin particles; sorption; oxyanion removal; melamine-formaldehyde resin; hard templating; silica; water treatment; selectivity.

Table of Content

1. Results ... 3
1.1. Characterization ... 3
1.1.1. FTIR spectra of the P-PMF and H-PMF samples ... 3
1.1.2. SEM images of the hybrid particles ... 5
1.1.3. N_2 sorption isotherm of Ludox® HS-40 ... 5
1.1.4. Pore size distribution of hybrid H-PMF particles and Ludox® HS-40 ... 6
1.1.5. Streamingpotential measurements ... 7
1.2. Adsorption Experiments ... 8
1.2.1. pH measurements for adsorption experiments with H-PMF-66 ... 8
1.2.2. pH measurements for adsorption experiments with P-PMF-66 ... 8
1.2.3. pH measurements for adsorption experiments with P-PMF-79 ... 9
1.2.4. pH measurements for adsorption experiments with P-PMF-85 ... 9
1.2.5. pH measurements for adsorption experiments with P-PMF-88 ... 10
1.2.6. pH measurements for adsorption experiments with P-PMF-89 ... 10
1.2.1. Fitting comparison ... 11
1.2.2. pH measurements for sulfate/phosphate selectivity experiments for all samples 13
1.2.3. pH measurements for sulfate/phosphate selectivity experiments onto H-PMF-66 14
1.3. Comparison of the obtained adsorption capacities for sulfate with other materials .. 15
References ... 17

1. Results

1.1. *Characterization*

1.1.1. FTIR spectra of the P-PMF and H-PMF samples

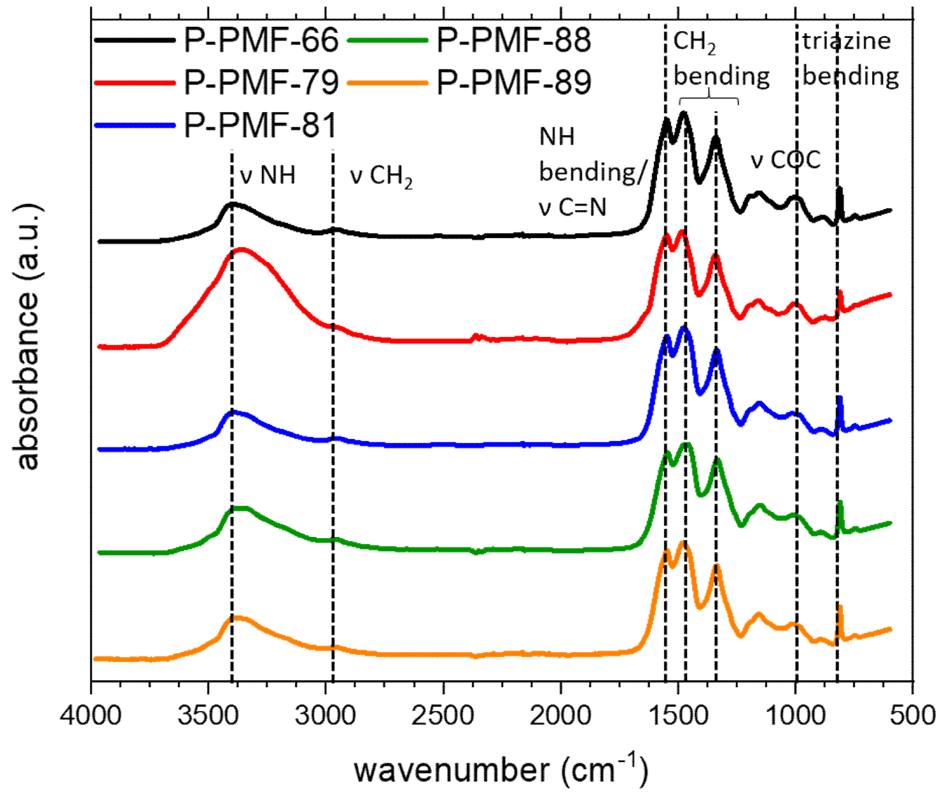

Figure S1. ATR-FTIR spectra of P-PMF with the predominant mode assignment and normalized to the triazine bending at 812 cm^{-1} for reasons of comparability.

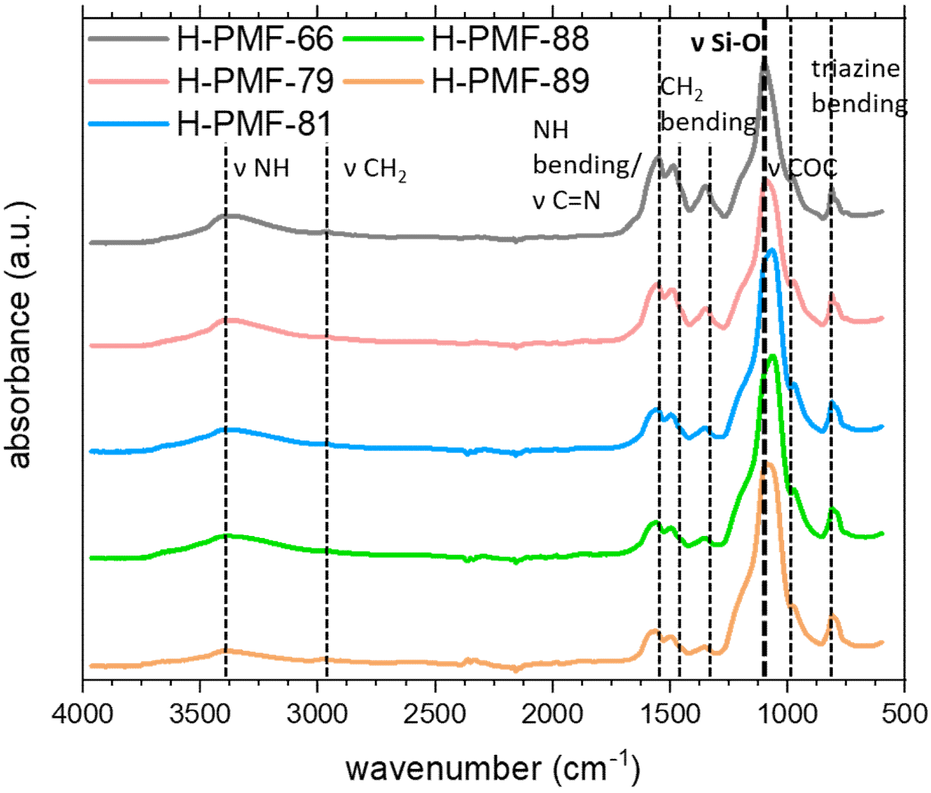

Figure S2. ATR-FTIR spectra of H-PMF with the predominant mode assignment and normalized to the triazine bending at 812 cm^{-1} for comparability.

1.1.2. SEM images of the hybrid particles

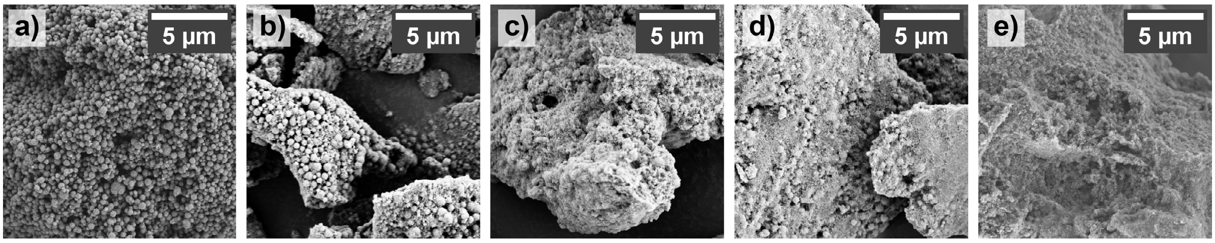

Figure S3. SEM images of a) H-PMF-66, b) H-PMF-79, c) H-PMF-85, d) H-PMF-88, and H-PMF-89.

1.1.3. N_2 sorption isotherm of Ludox® HS-40

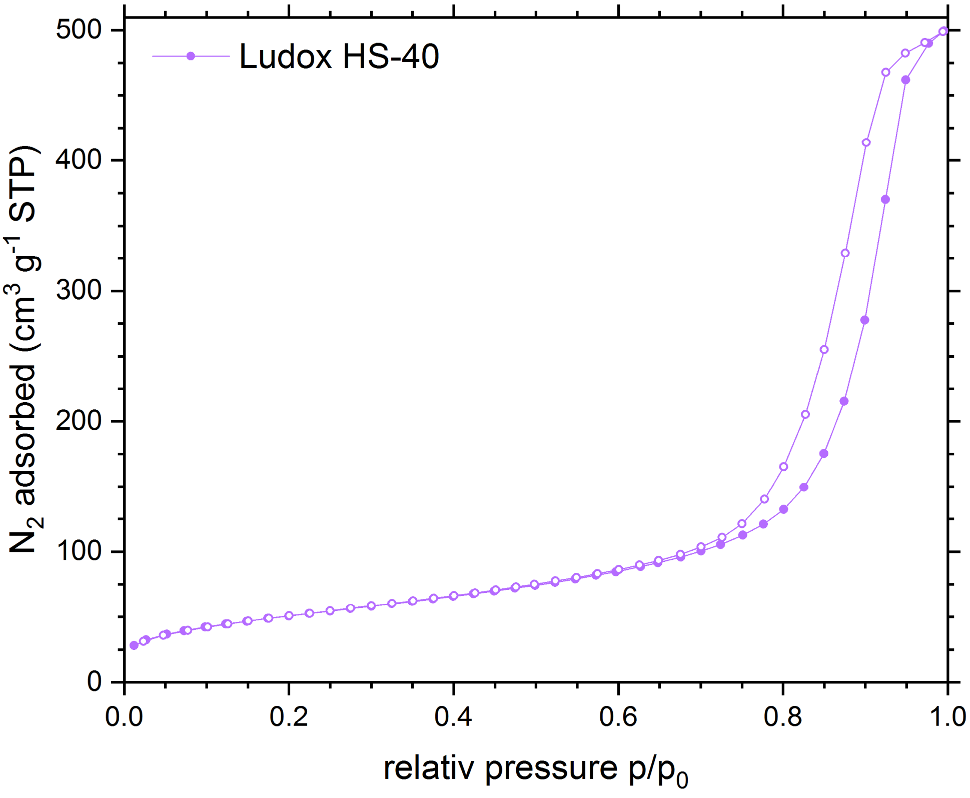

Figure S4. Nitrogen (N_2) de-/adsorption isotherms measured at 77 K for Ludox® HS-40. Data points in the adsorption and desorption branch of the isotherms are indicated by filled and empty symbols, respectively.

1.1.4. Pore size distribution of hybrid H-PMF particles and Ludox® HS-40

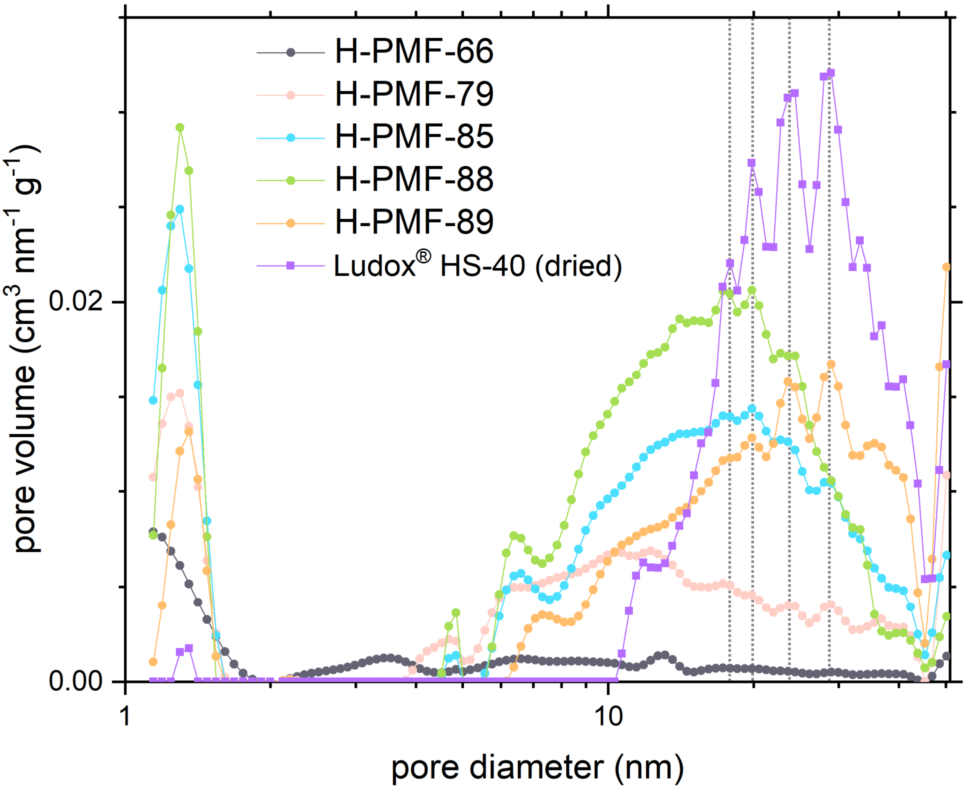

Figure S5. Pore size distribution (PSD) of H-PMF samples and Ludox® HS-40 (Quenched solid density functional theory fit; slit/cylindrical/sphere pores; adsorption branch). PMF-66 is shown in black, PMF-79 in red, PMF-81 in blue, PMF-88 in green, PMF-89 in orange and Ludox® HS-40 in violet. Main peaks of Ludox® HS-40 PSD marked with dashed grey line for comparison.

1.1.5. Streamingpotential measurements

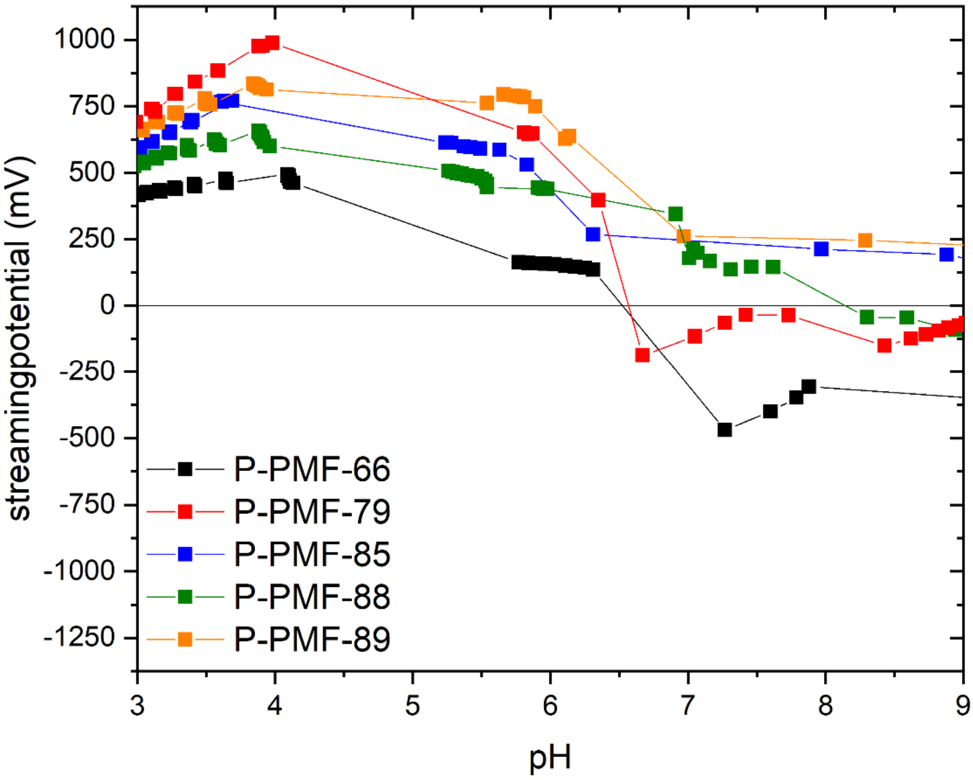

Figure S6. Streaming potential vs. pH curves of the P-PMF samples with P-PMF-66 (black), P-PMF-79 (red), P-PMF-85 (blue), P-PMF-88 (green) and P-PMF-89 (orange).

1.2. Adsorption Experiments

1.2.1. pH measurements for adsorption experiments with H-PMF-66

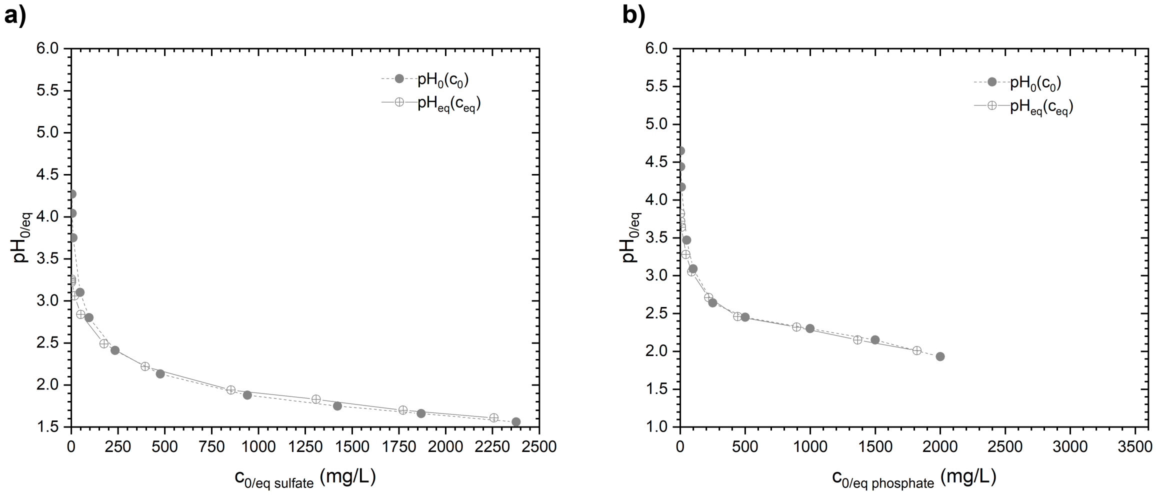

Figure S7. $pH_0(c_0)$ (filled symbols) and $pH_{eq}(c_{eq})$ (crossed symbols) points for the adsorption experiments of H-PMF-66 with a) H_2SO_4 and b) H_3PO_4.

1.2.2. pH measurements for adsorption experiments with P-PMF-66

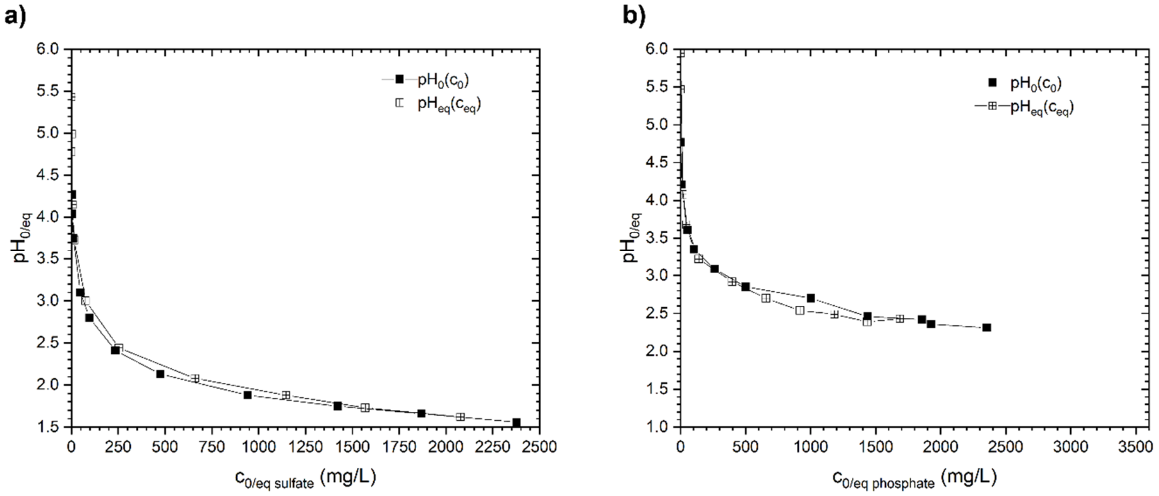

Figure S8. $pH_0(c_0)$ (filled symbols) and $pH_{eq}(c_{eq})$ (crossed symbols) points for the adsorption experiments of P-PMF-66 with a) H_2SO_4 and b) H_3PO_4.

1.2.3. pH measurements for adsorption experiments with P-PMF-79

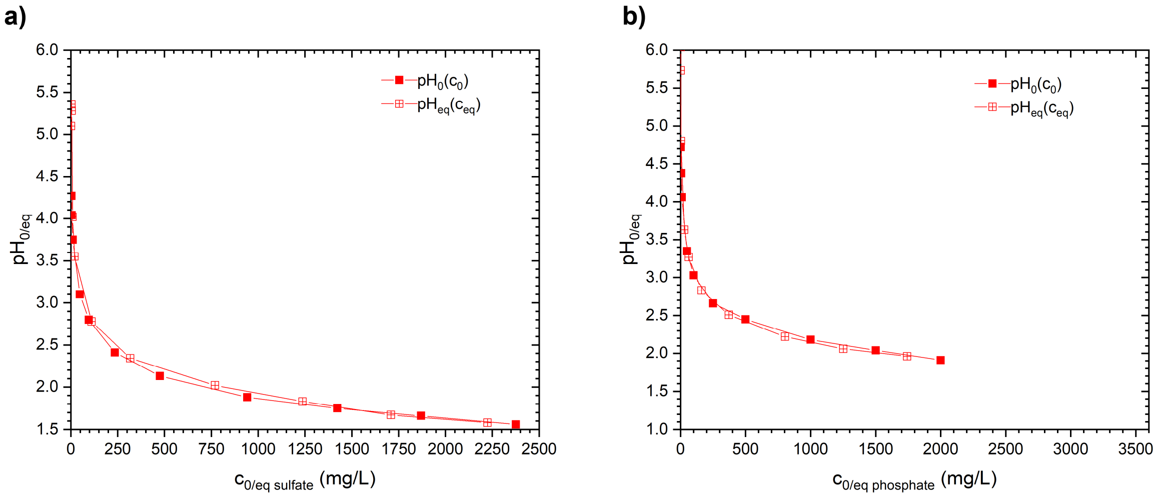

Figure S9. $pH_0(c_0)$ (filled symbols) and $pH_{eq}(c_{eq})$ (crossed symbols) points for the adsorption experiments of P-PMF-79 with a) H_2SO_4 and b) H_3PO_4.

1.2.4. pH measurements for adsorption experiments with P-PMF-85

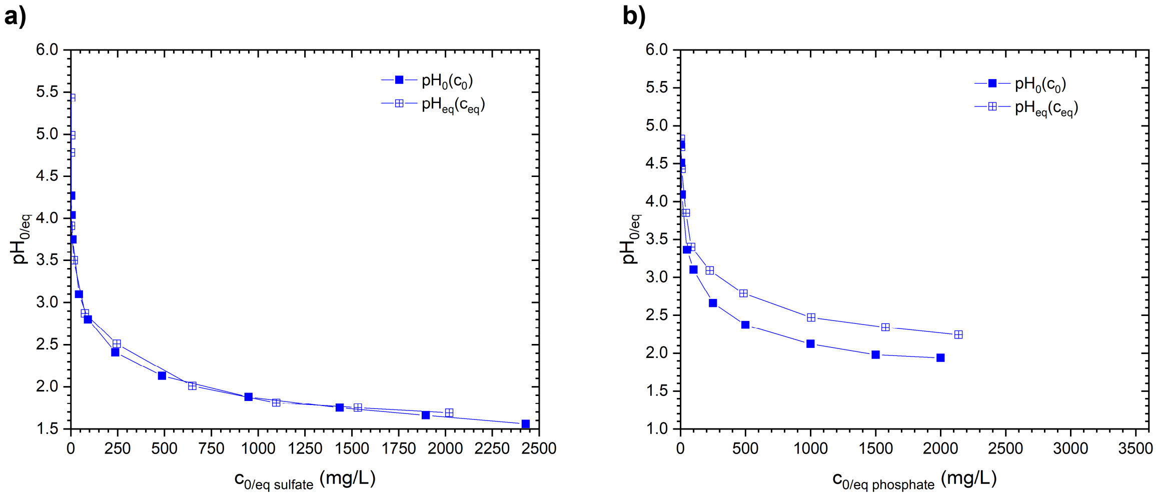

Figure S10. $pH_0(c_0)$ (filled symbols) and $pH_{eq}(c_{eq})$ (crossed symbols) points for the adsorption experiments of P-PMF-85 with a) H_2SO_4 and b) H_3PO_4.

1.2.5. pH measurements for adsorption experiments with P-PMF-88

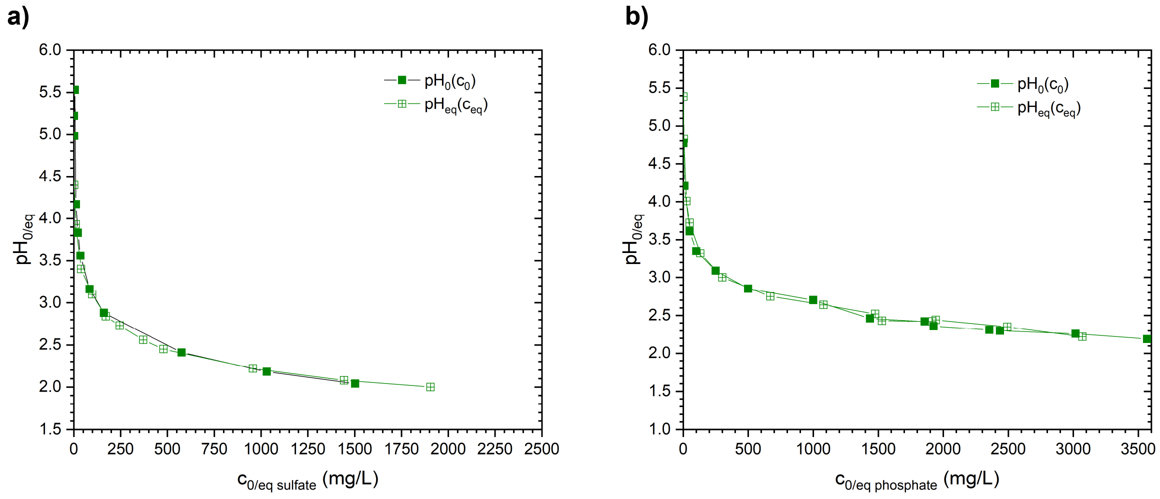

Figure S11. $pH_0(c_0)$ (filled symbols) and $pH_{eq}(c_{eq})$ (crossed symbols) points for the adsorption experiments of P-PMF-88 with a) H_2SO_4 and b) H_3PO_4.

1.2.6. pH measurements for adsorption experiments with P-PMF-89

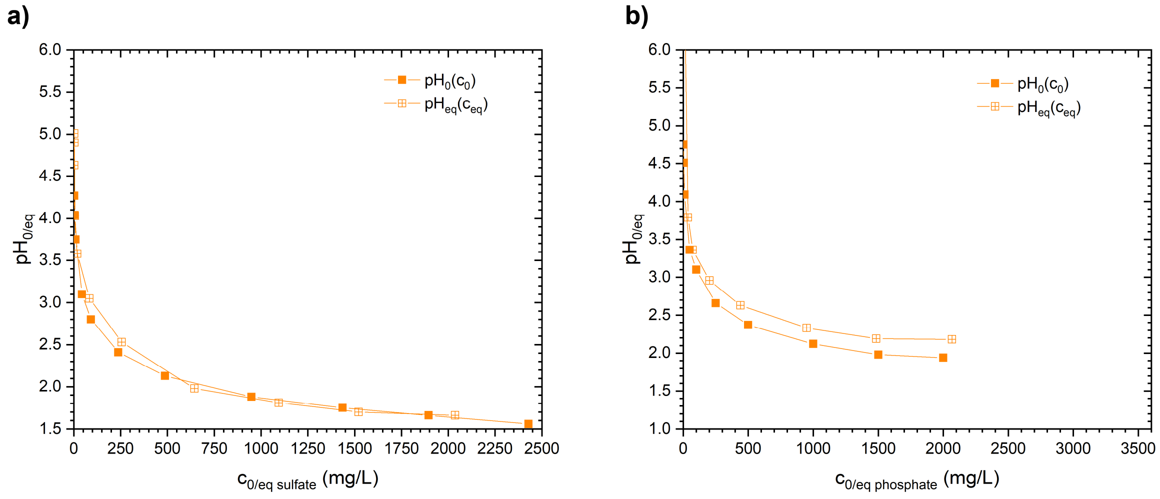

Figure S12. $pH_0(c_0)$ (filled symbols) and $pH_{eq}(c_{eq})$ (crossed symbols) points for the adsorption experiments of P-PMF-89 with a) H_2SO_4 and b) H_3PO_4.

1.2.1. Fitting comparison

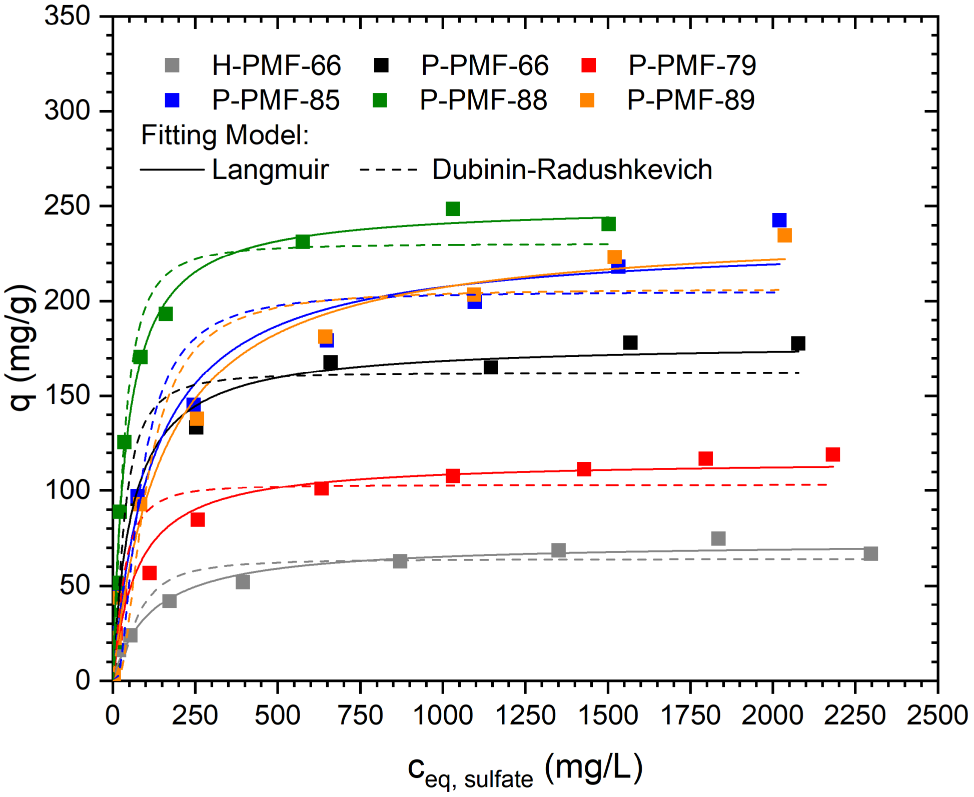

Figure S13. Sorption isotherms for sulfate ions onto H-PMF-66 (gray), P-PMF-66 (black), P-PMF-79 (red), P-PMF-85 (blue), P-PMF-88 (green) and P-PMF-89 (orange) with the corresponding Langmuir fits (solid lines) and Dubinin-Radushkevich fits (dashed line). The corresponding pH values are displayed in Figure S7 – S12. The fitting parameters are displayed in Table 4.

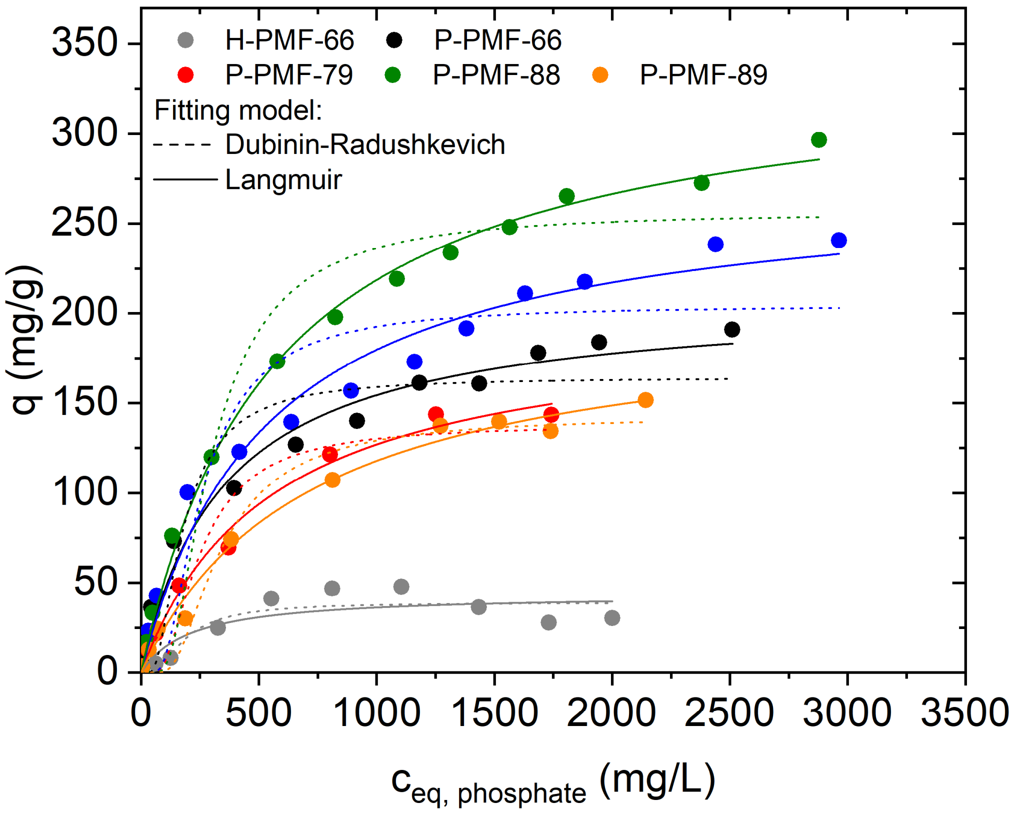

Figure S14. Sorption isotherms for phosphate ions onto H-PMF-66 (gray), P-PMF-66 (black), P-PMF-79 (red), P-PMF-85 (blue), P-PMF-88 (green) and P-PMF-89 (orange) with the corresponding Langmuir fits (solid lines) and Dubinin-Radushkevich fits (dashed line). The corresponding pH values are displayed in Figure S7 – S12. The fitting parameters are displayed in Table5.

1.2.2. pH measurements for sulfate/phosphate selectivity experiments for all samples

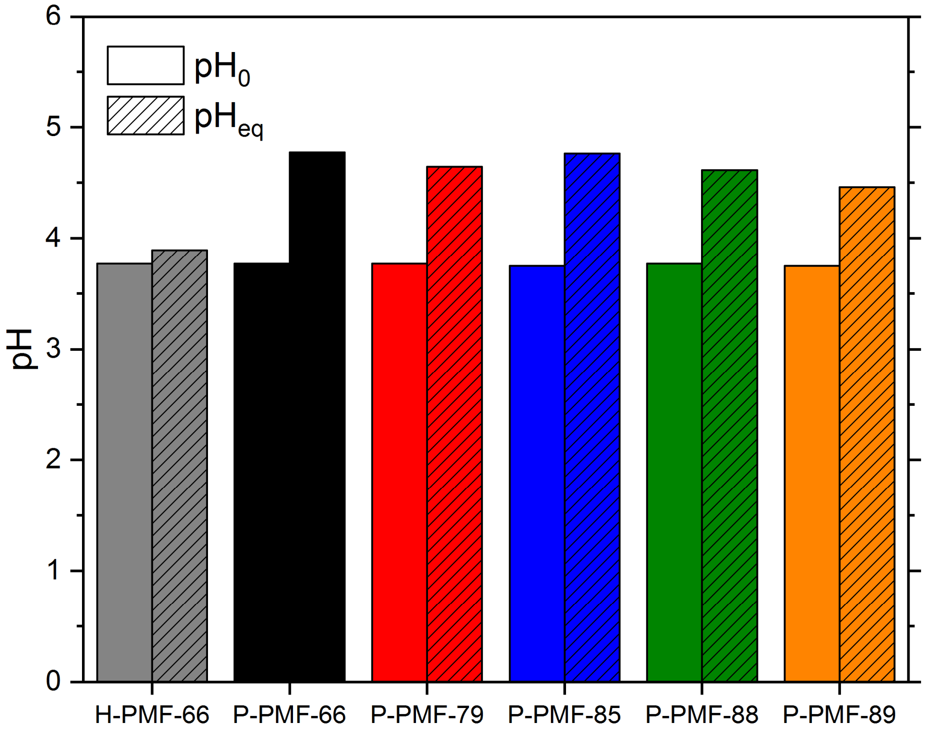

Figure S15. pH_0 (solid) and pH_{eq} (striped) values for the phosphate/sulfate selectivity adsorption experiments with a solution containing both, 10 mg/L PO_4^{3-} and 10 mg/L SO_4^{2-}, whereby H-PMF-66 is shown in gray, PMF-66 in black, P-PMF-79 in red, P-PMF-85 in blue, P-PMF-88 in green and P-PMF-89 in orange.

1.2.3. pH measurements for sulfate/phosphate selectivity experiments onto H-PMF-66

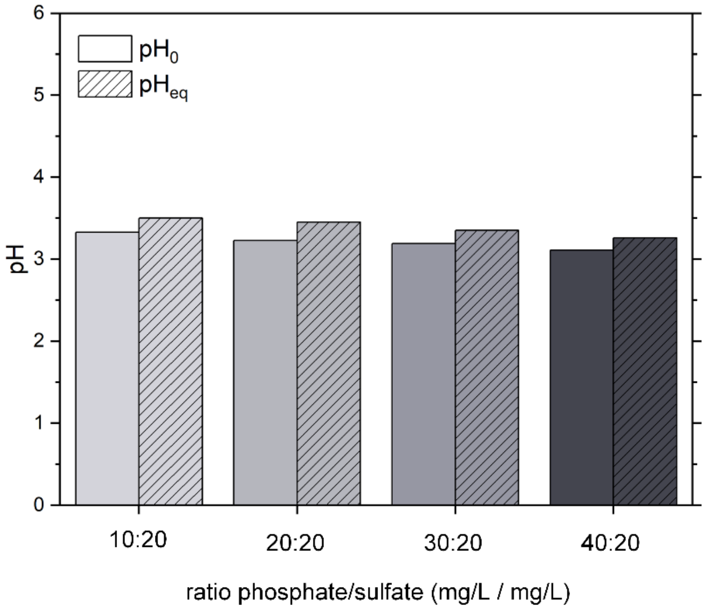

Figure S16. pH_0 (solid) and pH_{eq} (striped) values for the phosphate/sulfate selectivity adsorption experiments with a solution containing different concentrations of phosphate and 20 mg/L sulfate onto H-PMF-66 with pH_0 shown as solid bar and pH_{eq} shown as striped bar.

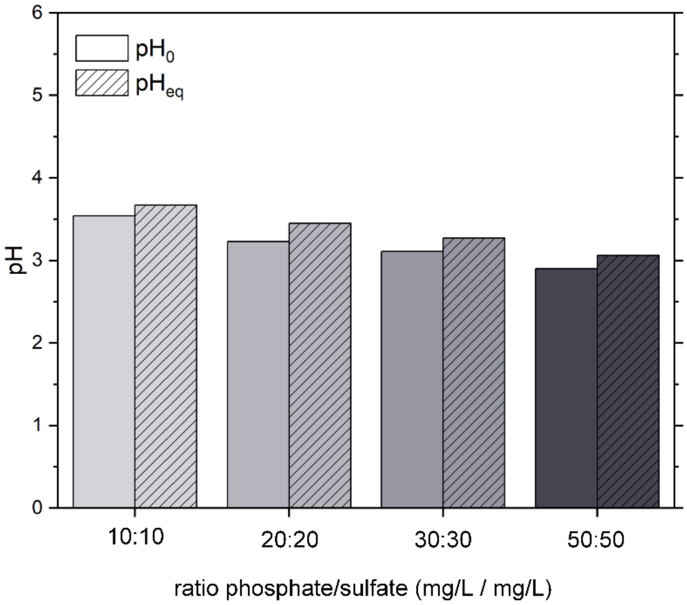

Figure S17. pH_0 (solid) and pH_{eq} (striped) values for the phosphate/sulfate selectivity adsorption experiments with a solution containing 1 : 1 ratios of phosphate and sulfate at different concentrations onto H-PMF-66 with pH_0 shown as solid bar and pH_{eq} shown as striped bar.

1.3. Comparison of the obtained adsorption capacities for sulfate with other materials

Table S1. Sorption capacities for the removal of sulfate compounds from aqueous solutions with different sorbent materials and adsorbent doses (a.d.). The obtained sorption capacities from this work were achieved in the batch adsorption experiments.

Material	Sorption capacity q (mg/g)	Compound	Experimental conditions			Ref.
			**pH_0**	**a.d. (g/L)**	**t, T**	
P-PMF-88	251	H_2SO_4	2.0	1.67	24 h, 25 °C	This work
P-PMF-89	238	H_2SO_4	1.6	1.67	24 h, 25 °C	This work
P-PMF-85	232	H_2SO_4	1.6	1.67	24 h, 25 °C	This work
P-PMF-66	178	H_2SO_4	1.6	1.67	24 h, 25 °C	This work
P-PMF-79	116	H_2SO_4	1.6	1.67	24 h, 25 °C	This work
H-PMF-66	73	H_2SO_4	1.6	1.67	24 h, 25 °C	This work
Amberlite IRA-900	56.5	Na_2SO_4	7.5	20	24 h, 20 °C	[1]
Lewatit K6362	166.7	n.d.	9	5	24 h, 25 °C	[2]

Table S2. Sorption capacities for removal of phosphate from aqueous solutions with different sorbent materials and adsorbent doses (a.d.). The obtained sorption capacities from this work were achieved in the batch adsorption experiments.

Material	Sorption capacity q (mg/g)	Compound	Experimental conditions			Ref.
			**pH_0**	**a.d. (g/L)**	**t, T**	
P-PMF-88	341	H_3PO_4	2.2	1.67	24 h, 25 °C	This work
P-PMF-85	275	H_3PO_4	2.0	1.67	24 h, 25 °C	This work
P-PMF-66	209	H_3PO_4	1.9	1.67	24 h, 25 °C	This work
P-PMF-89	201	H_3PO_4	1.9	1.67	24 h, 25 °C	This work
P-PMF-79	196	H_3PO_4	1.9	1.67	24 h, 25 °C	This work
H-PMF-66	44	H_3PO_4	1.9	1.67	24 h, 25 °C	This work
Duolite A 171	44.0	KH_2PO_4	5.0 - 5.5	n.d.	30 min, 25 °C	[3]
Purolite FerrIXA33E	147.2	KH_2PO_4	7.2	0.1 - 10	72 h, 24 °C	[4]
AMBERLITE™ HPR 9200	40.4	Na_2HPO_4	7.5	2	24 h, 25 °C	[5]
FerrIX™A33E	23.6	Na_2HPO_4	7.5	2	24 h, 25 °C	[5]
Zeolite	0.4	KH_2PO_4	n.d.	10	n.d., 20 °C	[6]

References

1. Tan, L.C.; Calix, E.M.; Rene, E.R.; Nancharaiah, Y.V.; van Hullebusch, E.D.; Lens, P.N.L. Amberlite IRA-900 Ion Exchange Resin for the Sorption of Selenate and Sulfate: Equilibrium, Kinetic, and Regeneration Studies. *J. Environ. Eng.* **2018**, *144*, 4018110, doi:10.1061/(ASCE)EE.1943-7870.0001453.
2. Haghsheno, R.; Mohebbi, A.; Hashemipour, H.; Sarrafi, A. Study of kinetic and fixed bed operation of removal of sulfate anions from an industrial wastewater by an anion exchange resin. *J. Hazard. Mater.* **2009**, *166*, 961–966, doi:10.1016/j.jhazmat.2008.12.009.
3. Sowmya, A.; Meenakshi, S. Removal of nitrate and phosphate anions from aqueous solutions using strong base anion exchange resin. *Desalination and Water Treatment* **2013**, *51*, 7145–7156, doi:10.1080/19443994.2013.771286.
4. Nur, T.; Johir, M.A.H.; Loganathan, P.; Nguyen, T.; Vigneswaran, S.; Kandasamy, J. Phosphate removal from water using an iron oxide impregnated strong base anion exchange resin. *Journal of Industrial and Engineering Chemistry* **2014**, *20*, 1301–1307, doi:10.1016/j.jiec.2013.07.009.
5. Li, Z.; Xu, S.; Li, Y.; Arai, Y. Novel Application of Hybrid Anion Exchange Resin for Phosphate Desorption Kinetics in Soils: Minimizing Re-Adsorption of Desorbed Ions. *Soil Syst.* **2020**, *4*, 36, doi:10.3390/soilsystems4020036.
6. Ma, J.; Lenhart, J.H.; Tracy, K. Orthophosphate Adsorption Equilibrium and Breakthrough on Filtration Media for Storm-Water Runoff Treatment. *J. Irrig. Drain Eng.* **2011**, *137*, 244–250, doi:10.1061/(ASCE)IR.1943-4774.0000291.
